# Candidate SNP markers of reproductive potential are predicted by a significant change in the affinity of TATA-binding protein for human gene promoters

**DOI:** 10.1186/s12864-018-4478-3

**Published:** 2018-02-09

**Authors:** Irina V. Chadaeva, Petr M. Ponomarenko, Dmitry A. Rasskazov, Ekaterina B. Sharypova, Elena V. Kashina, Dmitry A. Zhechev, Irina A. Drachkova, Olga V. Arkova, Ludmila K. Savinkova, Mikhail P. Ponomarenko, Nikolay A. Kolchanov, Ludmila V. Osadchuk, Alexandr V. Osadchuk

**Affiliations:** 1 Brain Neurobiology and Neurogenetics Center, Institute of Cytology and Genetics, Siberian Branch of Russian Academy of Sciences, 10 Lavrentyev Ave, Novosibirsk, 630090 Russia; 2 Novosibirsk State University, Novosibirsk, 630090 Russia; 3 Department of Biology, University of La Verne, La Verne, CA 91750 USA; 4 Vector-Best Inc., Koltsovo, Novosibirsk Region 630559 Russia; 5 Novosibirsk State Agricultural University, Novosibirsk, 630039 Russia

**Keywords:** Reproductive potential, Gene, Promoter, TATA box, TATA-binding protein, Single nucleotide polymorphism, SNP marker, Keyword-based search, Prediction, Verification

## Abstract

**Background:**

The progress of medicine, science, technology, education, and culture improves, year by year, quality of life and life expectancy of the populace. The modern human has a chance to further improve the quality and duration of his/her life and the lives of his/her loved ones by bringing their lifestyle in line with their sequenced individual genomes. With this in mind, one of genome-based developments at the junction of personalized medicine and bioinformatics will be considered in this work, where we used two Web services: (i) SNP_TATA_Comparator to search for alleles with a single nucleotide polymorphism (SNP) that alters the affinity of TATA-binding protein (TBP) for the TATA boxes of human gene promoters and (ii) PubMed to look for retrospective clinical reviews on changes in physiological indicators of reproductive potential in carriers of these alleles.

**Results:**

A total of 126 SNP markers of female reproductive potential, capable of altering the affinity of TBP for gene promoters, were found using the two above-mentioned Web services. For example, 10 candidate SNP markers of thrombosis (e.g., rs563763767) can cause overproduction of coagulation inducers. In pregnant women, Hughes syndrome provokes thrombosis with a fatal outcome although this syndrome can be diagnosed and eliminated even at the earliest stages of its development. Thus, in women carrying any of the above SNPs, preventive treatment of this syndrome before a planned pregnancy can reduce the risk of death. Similarly, seven SNP markers predicted here (e.g., rs774688955) can elevate the risk of myocardial infarction. In line with Bowles’ lifespan theory, women carrying any of these SNPs may modify their lifestyle to improve their longevity if they can take under advisement that risks of myocardial infarction increase with age of the mother, total number of pregnancies, in multiple pregnancies, pregnancies under the age of 20, hypertension, preeclampsia, menstrual cycle irregularity, and in women smokers.

**Conclusions:**

According to Bowles’ lifespan theory—which links reproductive potential, quality of life, and life expectancy—the above information was compiled for those who would like to reduce risks of diseases corresponding to alleles in own sequenced genomes. Candidate SNP markers can focus the clinical analysis of unannotated SNPs, after which they may become useful for people who would like to bring their lifestyle in line with their sequenced individual genomes.

**Electronic supplementary material:**

The online version of this article (10.1186/s12864-018-4478-3) contains supplementary material, which is available to authorized users.

## Background

Incessant progress in medical and biological sciences, advancement of technology, and education year in and year out improve quality of life and life expectancy of the population, creating comfortable conditions for active living. Nonetheless, there are numerous factors that adversely affect human health. They can include, for example, different kinds of environmental pollution, an increase in population density, which leads to the rapid spread of infections and parasitoses, and an increase in psychological stress. This situation not only reduces the quality of life and longevity of the individual but also has a deferred, long-term effect on the next generation, by acting as a mutagen [1]. The accumulating mutational load often worsens health and reduces the subsequent generation’s survival and adaptation to their habitat that ultimately reduces the chances of sustainable population reproduction.

The effects of the above factors limit individual reproductive potential: a concept used in population ecology to assess the evolutionary success of an individual [2] or a population [3]. In the 1970s, Eric Pianka defined reproductive potential as the most important conditional indicator reflecting a population’s ability to reproduce, survive, and develop under optimal ecological conditions [2–5]. In the context of human society, in the term “reproductive potential,” researchers can also include the mental state and physical state that allow a person to produce healthy offspring when social and physical maturity is achieved. Consequently, reproductive potential depends not only on physiological readiness for reproduction (primarily the reproductive system), but also on the general physical condition (with the exception of existing diseases that are incompatible with the implementation of reproduction) and on socio-economic status. With this in mind, everything is focused on individual ability for reproduction until the next generation becomes reproductive. In particular, not only the phenotype plays a role here, but so does the genotype, where most abilities of a given individual are encoded, both normal and mutational as well as epigenetic ones. It should also be noted that reproductive potential varies throughout the life cycle and does so in different ways for men and women. Ideally, the evaluation of reproductive potential would include not only the direct material and energy costs of reproduction but also the price of the risk associated with future reproductive attempts [5].

Predictive-preventive personalized medicine may help to improve individual reproductive success. Its methods include prediction (based on the analysis of the genome) of the probability of a specific disease, analysis of individual indicators, biomarkers (such as single nucleotide polymorphisms, SNPs [6, 7]), and the development of preventive and therapeutic measures for changing the physiological parameters of the reproductive potential in patients [8]. In particular, the analysis of SNP biomarkers allows a physician not only to make a prognosis for a patient regarding possible diseases that can reduce reproductive potential but also to adjust the prescribed treatment, taking into account individual characteristics and reactions to medicines.

In addition, according to Bowles’ lifespan theory [9], which links reproductive potential, quality of life, and life expectancy of an individual, it is possible timely to prevent diseases, which correspond to the alleles of the decoded genotype.

Within the framework of the biggest modern scientific project “1000 Genomes”, 10545 individual genomes have already been sequenced [10]. The “reference human genome” is publicly available via the Ensembl database [11] using the Web service UCSC Genome Browser [12]. A total of 100,877,027 SNPs have been experimentally identified and stored in the dbSNP database [6]. Database dbWGFP [13] containing 8.58 billion possible human whole-genome SNPs has already been created for accumulation of predictions, experimental data, clinical observations, and any other information relevant for biomedical analysis of individual genomes. For such an analysis, the most valuable biomedical SNP markers—within the framework of personalized medicine—are those that can differ between the individual human genomes of patients having some pathology and the reference human genome [14]. To find such markers, cohorts of patients with a given disease and healthy volunteers (as a control) are compared in a clinical study (e.g., [15]).

As far as human health is concerned, the clinical search for biomedical SNP markers is the only acceptable method. Nevertheless, it is so laborious and expensive that its application to all 8.58 billion potentially possible SNPs [13] and all known human pathologies is rather unlikely. Moreover, both Haldane’s dilemma [16] and Kimura’s theory of neutral evolution [17] independently predict that the absolute majority of SNPs in humans are neutral and do not affect health in any way; thus, it is unclear why it is necessary to verify them clinically. With this in mind, the mainstream clinical search for SNP markers of a given disease is currently limited by the simplest idea about heuristic handmade selection of candidate SNPs for clinical testing among unannotated SNPs on the basis of their location near the human genes that are already clinically associated with this disease (e.g., [18, 19]). Accordingly, computer-based preliminary analysis of unannotated SNPs can eliminate the absolute majority of neutral SNPs to make the clinical cohort-based search for biomedical SNP markers faster, cheaper, and more targeted [20]. There are many public Web services [21–38] that facilitate the computer-based search for candidate SNP markers using various similarity measures based on whole-genome data in health [39], after treatment [40], and during a disease [41] or infection [42] to eliminate unannotated SNPs that bear the least resemblance to known biomedical SNP markers (i.e. to eliminate the most probable neutral SNPs). The Central Limit Theorem predicts that the accuracy of this similarity-based elimination of unannotated neutral SNPs increases with the increase in the size and diversity of whole-genome data under study [43].

Now, the best accuracy of this mainstream search corresponds to SNPs in protein-coding regions of genes [44], i.e., SNPs that damage proteins [45] whose defects are uncorrectable by treatment or lifestyle changes. On the contrary, the worst accuracy of this kind of search is seen for regulatory SNPs [11], which alter concentrations of proteins without any damage to the proteins, and such problems are correctable by medication and lifestyle. The best balance between the predictability and biomedical usefulness corresponds to the regulatory SNPs between nucleotide positions -70 and –20 upstream of a transcription start site (TSS) [46, 47] where TATA-binding protein (TBP) binds to the promoter at the very beginning of transcription initiation. This TBP–promoter complex is obligatory for any TSSes because the TBP knockout model animals (TBP^−/−^) are always inviable since their development cannot proceed past the blastula stage because their maternal supply of TBP is exhausted [48, 49]. Moreover, the TBP–promoter affinity linearly correlates with the transcription magnitude of the human gene containing this promoter [50]. This notion has been repeatedly confirmed experimentally (for review, see [51]). The canonical form of the TBP-binding site (TATA box, synonyms: Hogness box and Goldberg-Hogness box [52]) is the best-studied regulatory element among human gene promoters [47].

In our previous studies, we developed public Web service SNP_TATA_Comparator (http://beehive.bionet.nsc.ru/cgi-bin/mgs/tatascan/start.pl) [53] and applied it to predict candidate SNP markers within TATA boxes of human genes associated with obesity [54], autoimmune diseases [55], chronopathology [56], aggressiveness [57, 58], Alzheimer’s disease [59], and efficacy of anticancer chemotherapy [60] (for review, see [20]). In the present work, we applied our Web service [53] in the same way to human reproductive potential as the most common concept of population ecology dealing with the evolutionary success of either individuals [2] or populations [3].

## Results

Tables 1, 2, 3, 4, 5, 6 and 7 present the results obtained by our Web service [53] for the 126 known and candidate reproductive-potential-related SNP markers in the TBP-binding sites of human gene promoters (see Methods: Supplementary Method, Additional file 1).

**Table 1:** Known and candidate SNP markers of tumors in reproductive organs

*Gene*	dbSNP [6] rel. 147 or see [Ref]	5′ flank	wt mut	3′ flank	K_D_, nM					Known diseases (SNP markers) or *hypothetical disease (candidate SNP markers)*	[Ref] or *[this work]*
					wt mut	Δ	Z	α	ρ		
*ESR2*	rs35036378	cctctcggtc	t *g*	ttaaaaggaa	6 *8*	↓	5	10^-3^	B	ESR2-deficient pT1 breast tumor needing tamoxifen prophylaxis against cancer	[61]
	rs766797386	ttaaaaggaa	g *t*	aaggggctta	6 *7*	↓	3	10^-2^	C	*(hypothetically) the same disease*	*[this work]*
*HSD17B1*	rs201739205	aggtgatatc	a *c*	agcccagagc	13 *18*	↓	5	10^-6^	A	higher risk of breast cancer	[64]
	rs201739205	agcaggtgat	a *t*	tcaagcccag	13 *35*	↓	18	10^-6^	A	*(hypothetically) the same disease*	*[this work]*
	rs748743528	gcaggtgata	t *c*	caagcccaga	13 *28*	↓	13	10^-6^	A		
	rs755636251	ggcgaagcag	g *t*	tgatatcaag	13 *11*	↑	2	0.05	D	*(hypothetically)* higher risk of breast cancer	[68]
*PGR*	rs10895068	gggagataaa	g *a*	gagccgcgtg	10 *6*	↑	8	10^-6^	A	endometrial cancer caused by the spurious TATA box and its TSS disbalancing both α and β isoforms of progesterone receptor	[65]
	rs544843047	*agtcgggaga*	*t* *c*	*aaaggagccg*	10 22	↓	14	10^-6^	A	*(hypothetically) health as the norm without the above-mentioned spurious TATA box*	*[this work]*
*GSTM3*	rs1332018	ccccttatgt	c *a*	gggtataaag	4 3	=	2	1	E	maternal “c” (Wb: TF-binding site damaged, not TATA box), elevates risk of a brain tumor in her child, renal cancer, and Alzheimer’s disease	[66, 67]
	rs200209906	gtataaagcc	c t,a	ctcccgctca	3.6 4.3	↓	2	1	E	*(hypothetically) the same disease and low risks of breast cancer in those who never drink alcohol and lesser Hg-resistance during reproduction*	*[this work], *[69]
	rs750789679	cgggtataaa	g c	cccctcccgc	3.6 4.5	↓	3	10^-2^	C		
	rs748231432	cccttatgtc	g c,t	ggtataaagc	3.6 3.0	↑	3	0.05	D	*(hypothetically) lower risk of a brain tumor in a child whose mother has “c”-allele of rs1332018*	*[this work], *[66]
	rs763859166	gggtataaag	c t	ccctcccgct	3.6 2.9	↑	3	10^-2^	C		

**Fig. 1:**
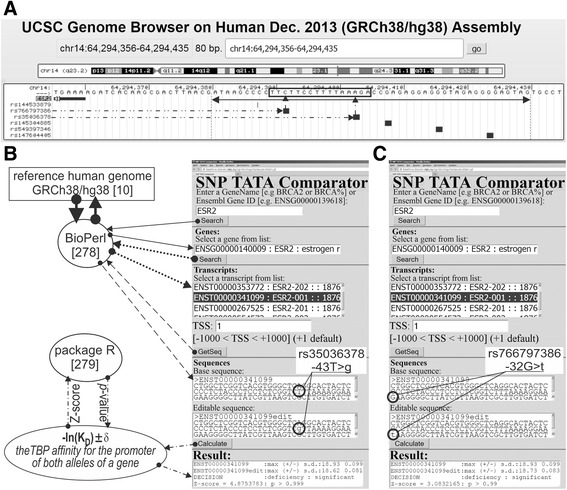
The result produced by SNP_TATA_Comparator [53] for reproductive potential-related SNP markers in the human *ESR2* gene. *Legend:* **a** Unannotated SNPs (analyzed in this study) in the region [-70; -20] (where all proven TBP-binding sites (boxed) are located; double-headed arrow, ↔) of the human *ESR2* gene promoter retrieved from dbSNP, rel. 147 [6] using the UCSC Genome Browser [12]. Dash-and-double-dot arrows: known and candidate SNP markers of reproductive potential are predicted by a significant change in the affinity of TBP for the human *ESR2* gene promoter. **b** and **c** The results from our Web service SNP_TATA_Comparator [53] for the two SNP markers of reproductive potential: known marker rs35036378 [61] and candidate marker rs766797386 near the known TBP-binding site (boxed) of the human *ESR2* gene promoter. Solid, dotted, and dashed arrows indicate queries in the reference human genome [10] by means of the BioPerl library [265]. Dash-and-dot arrows: estimates of significance of the alteration of gene product abundance in patients carrying the minor allele (mut) relative to the norm (ancestral allele, wt) expressed as a Z-score using package R [266]. Circles indicate the ancestral (wt) and minor (mut) alleles of the SNP marker labeled by its dbSNP ID [6]

Hereinafter, ancestral (wt) and minor (mut) alleles; K_D_, dissociation constant of TBP–DNA interaction; Δ, a change: overexpression (↑), deficit (↓), norm (=); α = 1 – p, significance {where p value is shown in Fig. 1; α = 1 denotes insignificance}; ρ, heuristic rank of candidate SNP markers varying in alphabetical order from the “best” (A) to the “worst” (E); the CETP gene: 18bp, the 18-bp deletion 5’-gggcggacatacatatac-3’; the F3 gene: 30bp, 17bp, and 18bp as the insertions 5’-agaccttcataagaaataatcctgatccaa-3’, 5’-tgctgcgtactggcaaa-3’, and 5’-acggcgtagagactggga-3’ of 30 bp, 17 bp, and 18 bp in length, respectively; EMSA, electrophoretic mobility shift assay; Hg, mercury; LUC, luciferase reporter assay; TF, transcription factor; Wb: western blot.

**Table 2:** Known and candidate SNP markers of tumors in nonreproductive organs

*Gene*	dbSNP [6] rel. 147 or see [Ref]	5′ flank	wt mut	3′ flank	K_D_, nM					Known diseases (SNP markers) or *hypothetical disease (candidate SNP markers)*	[Ref] or *[this work]*
					wt mut	Δ	Z	α	ρ		
*IL1B*	rs1143627	ttttgaaagc	c *t*	ataaaaacag	5 *2*	↑	15	10^-6^	A	high risks of gastric, liver, and non–small cell lung cancers; gastric ulcer, chronic gastritis, recurrent major depression, obesity, Graves’ disease, pre-eclampsia, *(hypothetically) short time-to-delivery in pregnancy and childbirth*	[77–85] *[this work]*
	rs549858786	tgaaagccat	a *t*	aaaacagcga	5 7	↓	8	10^-6^	A	*(hypothetically) lesser risk of the same diseases*	[60]
*CYP2A6*	rs28399433	tcaggcagta	t *g*	aaaggcaaac	2 *9*	↓	21	10^-6^	A	low risk of lung cancer in smokers (LUC: “-34g” corresponds to 50% of “-34t”), *(hypothetically) lesser damage from secondhand smoke in pregnant women who are nonsmokers*	[86, 87], *[this work],* [90–92]
	rs761592914	tttttcaggc	a *c*	gtataaaggc	2 *3*	↓	3	10^-3^	B	*(hypothetically) the same disease*	*[this work]*
*CYP2B6*	rs34223104	gatgaaattt	t *c*	ataacagggt	4 *10*	↓	15	10^-6^	A	TATA_WT_→USF_SNP,_ TSS_WT_→TATA_SNP_, and *de-novo* TSS_SNP_ can cause overexpression of this gene of a bioactivator of immunosuppressive and antitumor prodrug cyclophosphamide	[88]
	*rs563558831*	*tgaaatttta*	*t* *c*	*aacagggtgc*	4 *10*	↓	13	10^-6^	A	*(hypothetically) the same problem*	*[this work]*
*DHFR*	rs10168	ctgcacaaat	g *a*	gggacgaggg	15 *9*	↑	9	10^-6^	A	resistance to methotrexate treatment of leukemia and *(hypothetically) that in the cases of ectopic pregnancy, metastatic choriocarcinoma, and gestational trophoblastic disease*	[89], *[this work],* [93]
	rs750793297	tgcacaaatg	g *t*	ggacgagggg	15 *13*	↑	3	10^-2^	C	*(hypothetically) the same diseases*	*[this work]*
	rs766799008	ctgcacaaat	a *g*	tggggacgag	15 *19*	↓	3	10^-3^	B	*(hypothetically) greater bioactivity of methotrexate during treatment of leukemia, ectopic pregnancy, metastatic choriocarcinoma, and gestational trophoblastic disease*	*[this work],* [60]
	rs764508464	ctgcacaaat	a *-*	tggggacgag	15 *37*	↓	17	10^-6^	A		
	rs754122321	ctcgcctgca	c *g*	aaatggggac	15 *25*	↓	9	10^-3^	B		

See “Note” under Table 1

**Table 3:** Known and candidate reproductivity-related SNP markers in genes of hormones

*Gene*	dbSNP [6] rel. 147 or see [Ref]	5′ flank	wt mut	3′ flank	K_D_, nM					Known diseases (SNP markers) or *hypothetical disease (candidate SNP markers)*	[Ref] or *[this work]*
					wt mut	Δ	Z	α	ρ		
*LEP*	rs201381696	tcgggccgct	a *g*	taagaggggc	4 *12*	↓	17	10^-6^	A	*hypoleptinemia elevates risk of obesity*	[54, 106]
	rs200487063	tgatcgggcc	g *a*	ctataagagg	4 *2*	↑	6	10^-6^	A	*(hypothetically) hyperleptinemia elevates risk of hypertension in obesity*	*[this work],* [107, 110]
	rs34104384	ccgctataag	a *t*	ggggcgggca	4 *3*	↑	4	10^-2^	C		
*GCG*	rs183433761	gctggagagt	a *g*	tataaaagca	0.9 *1.6*	↓	17	10^-6^	A	resistance to obesity during a high-fat diet	[54]
										*(hypothetically) hypoglucogonemia decreases pregnancy probability, serum insulin in pregnancy, and during late gestational period*	*[this work], *[111, 112]
	rs757035851	tatataaaag	cag *-*	tgcgccttgg	0.9 *1.1*	↓	3	10^-3^	B		
*GH1*	rs11568827	aggggccagg	g *-*	tataaaaagg	1.5 *1.4*	=	1	1	E	short stature (EMSA: unknown TF-binding site lost, not TATA box)	[107]
										*(hypothetically) higher risk of GH1 deficiency as clinical syndrome whose symptoms are increased central adiposity, atherogenesis, as well as cerebrovascular and cardiac morbidity (and mortality), and, also, decreased lean body mass, bone mineral density, quality of life*	*[this work], *[113]
	rs796237787	gaaggggcca	g *-*	ggtataaaaa							
	rs768454929	agggtataaa	a *c*	agggcccaca	1.5 *2.6*	↓	7	10^-6^	A		
	rs761695685	gccagggtat	a *g*	aaaagggccc	1.5 *5.8*	↓	19	10^-6^	A		
	rs774326004	ccagggtata	a *t*	aaagggccca	1.5 *0.9*	↑	7	10^-6^	A	*(hypothetically) higher risks of acromegaly*	*[this work],* [114]
	rs777003420	aaggggccag	g *t*	gtataaaaag	1.5 *1.3*	↑	3	0.05	D		
*INS*	rs5505	agatcactgt	c *t*	cttctgccat	53 *44*	↑	4	10^-3^	B	type 1 diabetes after neonatal diabetes mellitus	[108]
										*(hypothetically) hyperinsulinemia elevates the placental leptin which causes neonatal macrosomia*	*[this work], *[115]
	rs563207167	tcagccctgc	c *t*	tgtctcccag	53 *44*	↑	4	10^-3^	B		
	rs11557611	gatcactgtc	c *t*	ttctgccatg	53 *60*	↓	2	0.05	D	*(hypothetically) hypoinsulinemia slows down fetal growth*	*[this work], *[116]

See “Note” under Table 1

**Table 4:** Known and candidate reproductivity-related SNP markers in genes of other metabolic proteins

*Gene*	dbSNP [6] rel. 147 or see [Ref]	5′ flank	wt mut	3′ flank	K_D_, nM					Known diseases (SNP markers) or *hypothetical disease (candidate SNP markers)*	[Ref] or *[this work]*
					wt mut	Δ	Z	α	ρ		
*NOS2*	-51t→c [288]	gtataaatac	t *c*	tcttggctgc	2 *1*	↑	3	10^-2^	C	resistance to malaria and epilepsy *(hypothetically) higher risk of gestational diabetes mellitus*	[288–290], *[this work],* [147]
*STAR*	rs16887226	cagccttcag	c *t*	gggggacatt	10 10	=	0	1	E	hypertensive diabetic patients, (EMSA: unknown TF-binding site lost rather than TATA box)	[291]
	rs544850971	*tcagcggggg*	*a* *g*	*catttaagac*	10 12	↓	5	10^-2^	C	(*hypothetically) lower risk of the same disease and congenital adrenal hyperplasia*	*[this work], *[148]
*APOA1*	35a→c [292]	tgcagacata	a *c*	ataggccctg	3 *4*	↓	5	10^-6^	A	fatty liver *(hypothetically) high risk of polycystic ovary syndrome in young women*	[292], *[this work], *[149]
*CETP*	DEL-51(18 bp) [293]	cgtgggggct	18bp -	gggctccagg	4 *7*	↓	7	10^-6^	A	hyperalphalipoproteinemia reducing risk of atherosclerosis	[293]
	rs17231520	ggggctgggc	g *a*	gacatacata	4 *2*	↑	10	10^-6^	A	*(hypothetically) biomarker of late pregnancy when plasma triglyceride, high-density lipoprotein, and cholesterol concentrations are significantly increased*	*[this work],* [150]
	rs569033466	atacatatac	g *a*	ggctccaggc	4 *3*	↑	4	10^-3^	B		
	rs757176551	catatacggg	c *g*	tccaggctga	4 *2*	*↑*	*10*	10^-6^	A		
*SOD1*	rs7277748	ggtctggcct	a *g*	taaagtagtc	2 *7*	↓	17	10^-6^	A	amyotrophic lateral sclerosis, *(hypothetically), asthenospermia, lower female fertility via progesterone deficiency*	[294], *[this work],* [151, 152]
*TPI1*	rs1800202	gcgctctata	t g	aagtgggcag	1 4	↓	17	10^-6^	B	hemolytic anemia, neuromuscular diseases	[295, 296]
										*(hypothtically) higher risk of asthenospermia*	*[this work],* [153]
	rs781835924	cgcggcgctc	t c	atataagtgg	1 2	↓	10	10^-6^	B		
*GJA5*	rs10465885	caactaagat	g a	tattaaacac	3 3	=	1	1	E	arrhythmia, cardiovascular events (LUC: TF-binding site damaged, not TATA box)	[297]
										*(hypothetically) the same disease and higher risk of heart morphogenesis disorders*	*[this work], *[154]
	rs587745372	ggcgacagat	a t	cgattaaaaa	6 7	*↓*	*3*	10^-3^	B		
	rs35594137	gaggagggaa	g a	gcgacagata	6 6	=	0	1	E		
										arrhythmia, cardiovascular events (LUC: TF-binding site damaged, not TATA box)	[298]

See “Note” under Table 1

**Table 5:** Known and candidate reproductivity-related SNP markers related to blood proteins

*Gene*	dbSNP [6] rel. 147 or see [Ref]	5′ flank	wt mut	3′ flank	K_D_, nM					Known diseases (SNP markers) or *hypothetical disease (candidate SNP markers)*	[Ref] or *[this work]*
					wt mut	Δ	Z	α	ρ		
*HBB*	rs397509430	gggctgggca	t *-*	atacaacagt	5 *29*	↓	34	10^-6^	A	malaria resistance and thalassemia	[176]
	rs33980857	gggctgggca	t *a,g,c*	atacaacagt	5 *21*	↓	27	10^-6^	A		
	rs34598529	ggctgggcat	a *g*	aaagtcaggg	5 *18*	↓	24	10^-6^	A		
	rs33931746	gctgggcata	a *g,c*	aagtcagggc	5 *11*	↓	14	10^-6^	A		
	rs33981098	agggctgggc	a *g,c*	taaaagtcag	5 *9*	↓	10	10^-6^	A		
	rs34500389	cagggctggg	c *a,t,g*	ataaaagtca	5 *6*	↓	3	10^-2^	C		
										*(hypothetically) the same disease; heterozygotes “wt-mut” are still more viable according to most of clinical indicators in comparison with both homozygotes “wt-wt” and “mut-mut”*	*[this work],* [185]
	rs63750953	ctgggcataa	aa -	gtcagggcag	5 *8*	*↓*	*9*	*10* ^*-6*^	A		
	rs281864525	tgggcataaa	a c	gtcagggcag	5 *7*	*↓*	*7*	*10* ^*-6*^	A		
	rs117785782	ggctgagggt	t c	tgaagtccaa	28 *39*	*↓*	*7*	*10* ^*-6*^	A		
*HBD*	rs35518301	caggaccagc	a *g*	taaaaggcag	4 *8*	↓	11	10^-6^	A	malaria resistance and thalassemia	[176]
										*(hypothetically) the same disease; heterozygotes “wt-mut” are still more viable according to most of clinical indicators*	*[this work], *[185]
	rs34166473	aggaccagca	t *c*	aaaaggcagg	4 *8*	*↓*	*18*	*10* ^*-6*^	A		
*HBG2*	rs745580140	ggagttgctc	ta *-*	cacaagctct	11 *22*	*↓*	*10*	*10* ^*-6*^	A		
*ACKR1*	rs2814778	ttggctctta	t *c*	cttggaagca	10 *12*	↓	4	10^-3^	B	low white-blood-cell count and resistance to malaria, *(hypothetically) pre-eclampsia*	[177, 178], *[this work],* [186]
*MBL2*	rs72661131	tctatttcta	t *c*	atagcctgca	2 *4*	↓	12	10^-6^	A	variable immunedefici-ency, pre-eclampsia, stroke,	[190–192]
										*(hypothetically) the same disease; higher risks of recurrent vulvovaginal infections*	*[this work], *[187]
	rs562962093	atctatttct	a *g*	tatagcctgc	2 *5*	↓	15	10^-6^	A		
	rs567653539	tttctatata	g *a*	cctgcaccca	2 *1*	↑	12	10^-6^	A	*(hypothetically) reduced risks of recurrent vulvovaginal infections*	
*MMP12*	rs2276109	gatatcaact	a *g*	tgagtcactc	11 *14*	↓	3	10^-2^	C	lower risk of psoriasis, systemic sclerosis, asthma	[193–195]
										*(hypothetically), higher risk of ovarian hyper-stimulation syndrome*	*[this work], *[188]
	rs572527200	gatgatatca	a *g*	ctatgagtca	11 *14*	↓	3	10^-2^	C		
*F2*	rs564528021	agttcaacat	t *c*	aacccagagg	13 *9*	↑	7	10^-6^	A	*(hypothetically) high risk of pre-eclampsia*	*[this work], *[189]
	rs752364393	caacattaac	c *t*	cagaggggtc	13 *11*	↑	3	10^-3^	B		

See “Note” under Table 1

**Table 6:** Known and candidate reproductivity-related SNP markers related to coagulation of blood

*Gene*	dbSNP [6] rel. 147 or see [Ref]	5′ flank	wt mut	3′ flank	K_D_, nM					Known diseases (SNP markers) or *hypothetical disease (candidate SNP markers)*	[Ref] or *[this work]*
					wt mut	Δ	Z	α	ρ		
*PROC*	rs528817178	cctttcattc	c t	gcttccacct	27 21	↑	5	10^-6^	A	*(hypothetically) higher risk of tumor cell invasion*	*[this work], *[214]
	rs539608065	ctttcattcc	g a	cttccacctg	27 22	↑	4	10^-3^	B		
	rs539731824	ttgtggttat	g a	gattaactcg	10 6	↑	8	10^-6^	A		
	rs756414294	ggcgcggcac	c t	agcaccagct	121 27	↑	25	10^-6^	A		
	rs777687270	ggcaccagca	c t	cagctgcccg	121 59	↑	13	10^-6^	A		
	rs746382956	tgcccgcaga	g a	gtgagcttcc	121 44	↑	19	10^-6^	A		
	rs542626506	cacacaggga	c t	agccctttca	27 31	↓	3	10^-2^	C	*(hypothetically) high risks of thrombosis, inflammation, and pregnancy loss*	*[this work], *[215]
	rs61731661	ccctttcatt	c t	cgcttccacc	27 29	↓	5	0.05	D		
*F8*	rs781855957	acggcggcag	c t	ggaagaggga	75 49	↑	8	10^-6^	A	*(hypothetically) higher risk of thrombosis*	*[this work] *[216]
*THBD*	rs13306848	agggagggcc	g *a*	ggcacttata	2 2	=	1	1	E	thrombosis (LUC: TF site damaged, not TATA)	[211]
										*(hypothetically) higher risks of placental failure and fetal loss*	*[this work], *[217]
	rs568801899	caatccgagt	g *a*	tgcggcatca	45 70	↓	6	10^-6^	A		
*F3*	rs563763767	ccctttatag	c *t*	gcgcggggca	3 *2*	↑	6	10^-6^	A	myocardial infarction; thrombosis;	[212]
										*(hypothetically) higher risk of ovarian cancer*	*[this work], *[218]
	rs779755900	atctcgccgc	- *30bp*	caactggtag	90 *10*	↑	43	10^-6^	A		
	rs749456955	gatctcgccg	c *a*	caactggtag	90 *75*	↑	4	10^-3^	B		
	rs746842194	cgatctcgcc	- *17bp*	gccaactggt	90 *31*	↑	15	10^-6^	A		
	rs754815577	ctcgatctcg	- *18bp*	ccgccaactg	90 *32*	↑	17	10^-6^	A		
	rs768753666	ggaacccgct	c *g*	gatctcgccg	90 *117*	↓	5	10^-6^	A	*(hypothetically) lower risk of ovarian cancer*	
	rs774688955	cgccacggaa	c *t*	ccgctcgatc	90 *101*	↓	2	0.05	D		
*F7*	-33a→c [213]	ccttggaggc	a *c*	gagaactttg	53 *62*	↓	3	10^-2^	C	moderate bleeding	[213]
										*(hypothetically) lower risk of ovarian cancer*	*[this work], *[218]
	rs749691733	agaactttgc	c *t*	cgtcagtccc	53 *66*	↓	4	10^-3^	B		
	*rs367732974*	aactttgccc	g *a*	tcagtcccat	53 *47*	↑	2	0.05	D	*(hypothetically) higher risk of ovarian cancer*	
	*rs549591993*	gcccgtcagt	c *a*	ccatggggaa	53 *25*	↑	13	10^-6^	A		
	rs777947114	agagaacttt	g *a*	cccgtcagtc	53 *19*	*↑*	*19*	10^-6^	A		
	rs770113559	gtcacccttg	g *a*	aggcagagaa	53 *41*	*↑*	*5*	10^-6^	A		
	rs754814507	cctcccccat	c *t*	cctctgtcac	53 *45*	*↑*	*3*	10^-3^	B		
*F11*	rs754739433	tctgggaatt	a g	tttttagtaa	4 5	↓	2	0.05	D	*(hypothetically) hereditary factor XI deficiency, high risk of spontaneous primary hemorrhage*	*[this work], *[219]
	rs780731761	ttatttttag	t a	aaaggaaatt	4 7	↑	8	10^-6^	A		
	rs747652067	tatttttagt	a g	aaggaaattt	4 7	↑	9	10^-6^	A		
	rs374761594	catttgtcta	c t	tgaagcacac	13 10	↑	3	10^-3^	B	*(hypothetically) higher risk of angioneurotic edema*	*[this work], *[220]
	rs759231858	acaccaacca	g t	aataacgaag	13 4	↑	17	10^-6^	A		
	rs752308147	ccagaataac	g a	aagctcgata	13 9	↑	6	10^-6^	A		
*F9*	rs371045754	tggtacaact	a c	atcgacctta	6 10	↓	5	10^-6^	A	*(hypothetically) higher risk of hemophilia B*	*[this work], *[221]
	rs750827465	tttggtacaa	c t	taatcgacct	6 4	↑	7	10^-6^	A	*(hypothetically) higher risk of myocardial fibrosis*	*[this work], *[222]

See “Note” under Table 1

**Table 7:** Candidate SNP markers of reproductivity-related genes

*Gene*	dbSNP [6] rel. 147 or see [Ref]	5′ flank	wt mut	3′ flank	K_D_, nM					*hypothetical disease (candidate SNP markers)*	*[this work],* [Ref]
					wt mut	Δ	Z	α	ρ		
*AR*	rs763353257	aagggaagta	g -	gtggaagatt	30 21	*↑*	6	10^-6^	A	*(hypothetically) higher risks of androgenetic alopecia and androgen-induced premature senescence in adult men*	[251]
	rs749306567	aagggaagta	g a	gtggaagatt	30 15	*↑*	11	10^-6^	A		
	rs377711437	cagcactgca	g a	ccacgacccg	75 66	*↑*	2	0.05	D		
*MTHFR*	rs780207553	cacgcactct	g a	ggcctgagct	74 38	*↑*	12	10^-6^	A	*(hypothetically) higher risk of pre-eclampsia*	[252]
	rs749532075	tccctcccca	c t^*)^	gcactctggg	74 50	*↑*	7	10^-6^	A		
	rs771960561	cctctgttcc	c t	tccccacgca	74 66	*↑*	3	10^-2^	B		
	rs773214376	tgcctctgtt	c t	cctccccacg	74 66	*↑*	2	0.05	D		
	rs566478202	ggtgcctctg	t g	tccctcccca	74 85	*↓*	2	0.05	D	*(hypothetically) higher risk of adverse pregnancy outcomes*	[253]
	rs752181249	gaggatctac	a c	gccatcagct	27 35	*↓*	4	10^-3^	B		
*DNMT1*	rs570287204	gtgggggggg	- gtg	tgtgtgcccg	52 23	*↑*	11	10^-6^	A	*(hypothetically) under stress, higher risk of epigenetic disorders of fetal and newborn brain development causing long-term neurobehavioral problems that may be reversible in adolescence*	[254, 255]
	rs534819409	cgtggggggg	g t	ggcctgagct	52 30	*↑*	7	10^-6^	A		
	rs553454792	gcgtgggggg	g t	gtgtgtgccc	52 23	*↑*	11	10^-6^	A		
	rs558447661	cgtggagctt	g t	gacgagccca	72 29	*↑*	15	10^-6^	A		
	rs535899986	cccagcaaac	c t	gtggagcttg	72 58	*↑*	5	10^-3^	B		
	rs143796354	cacctcccag	c a	aaaccgtgga	72 26	*↑*	20	10^-6^	A		
	rs756103340	gcggcgcgca	g a	cggcagttgg	92 79	*↑*	3	10^-3^	B		
	rs758026532	ccagcaaacc	g t^*)^	tggagcttgg	72 88	*↓*	4	10^-3^	B	*(hypothetically) higher risks of activation of protooncogenes in cancer*	[256]
	rs772821225	gtctccaata	a c	atgcagctgg	7 8	*↓*	2	0.05	D		
*CYP17A1*	rs758657961	ctggagttga	g a	ccagcccttg	56 30	*↑*	11	10^-6^	A	*(hypothetically) higher risk of hyperandrogenism in polycystic ovary syndrome*	[257]
	rs373488849	tgccctggag	t c	tgagccagcc	56 70	*↓*	4	10^-3^	B	*(hypothetically) higher risk of fertility impairments*	[258]
*NR5A1*	rs147497093	gttcagcaag	c t	acaagagaaa	19 6	*↑*	17	10^-6^	A	*(hypothetically) higher risks of adrenal tumors and endometriosis*	[259]
	rs535432539	cgctgcttcc	g a	cttcgtaagt	31 18	*↑*	9	10^-6^	A		
	rs553326158	gcgctgcttc	c t	gcttcgtaag	31 26	*↑*	3	10^-2^	C		
	rs143242438	caccctcatc	c t	ggtgtgagag	31 21	*↑*	6	10^-6^	A		

See the footnote of Table 1; ^*)^this SNP includes one more minor neutral allele: “a.”

First, we analyzed all SNPs mapped within [−70; −20] regions upstream of transcription start sites for the human genes containing the known biomedical SNP markers that alter TBP’s binding to promoters of these genes (Tables 1, 2, 3, 4, 5 and 6). Let us first describe in more detail only one human gene in order to briefly review all the others.

### Known and candidate reproductivity-related SNP markers of cancers

**The human** ***ESR2*** **gene** (estrogen receptor β) contains a known SNP marker (Fig. 1a: rs35036378) of an ESR2-deficient primary pT1 breast tumor, which is needed in tamoxifen-based prophylaxis of cancer [61] as shown in Table 1. The prediction of our Web service [53] is consistent with this independent clinical observation (Fig. 1b: text box “Results”, line “Decision” contains the label “deficiency: significant”).

Next, near this known biomedical SNP marker rs35036378, we found the unannotated SNP rs766797386, which can also decrease expression of the human *ESR2* gene (Fig. 1c) and thus cause an ESR2-deficient primary pT1 tumor requiring prophylaxis by tamoxifen against breast cancer [61]. This result allowed us to suggest rs766797386 as a candidate SNP marker of a higher risk of breast cancer reducing reproductive potential.

Finally, using our secondary keyword search for these two SNP markers (hereinafter: see Methods: Additional file 2: Figure S1. dotted-line box, Additional file 2), we learned (hereinafter: see Table S1, Additional file 3) that cadmium (Cd) elevates the risk of a primary tumor’s becoming malignant [62], whereas mothers undergoing tamoxifen-based treatment should not breastfeed [63].

The human *HSD17B1*, *PGR*, and *GSTM3* genes encode hydroxysteroid (17-β) dehydrogenase 1, progesterone receptor, and glutathione S-transferase μ3, respectively. Their promoters have the known SNP markers rs201739205, rs10895068, and rs1332018, which elevate risks of breast [64] and endometrial [65] cancers; a brain tumor in a fetus, newborn, or a child [66], respectively; as well as renal cancer and Alzheimer’s disease [67] (Table 1). Near these known biomedical SNP markers, there are four unannotated SNPs rs201739205, rs748743528, rs200209906, and rs750789679, which can similarly alter expression levels of the same genes according to the predictions of our Web service [53] (Table 1). Hence, we proposed them as the candidate SNP markers of the same diseases.

Besides, within the same promoters, we found four other unannotated SNPs rs755636251, rs544843047, rs748231432, and rs763859166, which can cause the opposite alterations in the expression of the corresponding genes (Table 1). Using our primary keyword search (hereinafter: see Methods, Additional file 2: Figure S1. two dashed-line boxes, Additional file 2), we found that both HSD17B1 overexpression and deficiency can elevate the risk of breast cancer [68], whereas GSTM3 deficiency can reduce these risks in people who never drink alcohol [69] (Table 1). In addition, Searles Nielsen and colleagues [66] suggested that another mechanism of GSTM3 overexpression can reduce the risk of a brain tumor in some children, as can rs748231432 and rs763859166 according to our results shown in Table 1.

Finally, using our secondary keyword search, we found eight retrospective clinical reviews [70–76]. The most interesting among them, in our opinion, is a report on a nontrivial balance between reproductive potential and the risk of cancers of reproductive organs [70]. It is interesting that only one SNP marker (rs605059; protein-coding region, *HSD17B1*) of a positive correlation between the lifespan and number of children in women is known so far [71]. It is also noteworthy that one of current theories is that aging is a stepwise reduction in reproductive potential of individuals where one of these steps is under the control of the luteinizing hormone, whose suppression by smoking can reduce the risk of Alzheimer’s disease [9].

The human *IL1B*, *CYP2A6*, *CYP2B6*, and *DHFR* genes encode interleukin 1β, xenobiotic monooxygenase, 1,4-cineole 2-exo-monooxygenase, and dihydrofolate reductase, respectively. Their promoters contain the known SNP markers (rs1143627 [77–85], rs28399433 [86, 87]) of nonreproductive organ cancer, as well as SNP markers (rs34223104 [88] and rs10168 [89]) of bioactivation and resistance to anticancer drugs, as shown in Table 2. Near these known SNP markers, we detected three unannotated SNPs, rs761592914, rs563558831, and rs750793297, which can alter expression levels of the same genes in the same manner (Table 2) and may be candidate SNP markers in this regard.

In addition, in the same gene regions, we found four other unannotated SNPs rs549858786, rs766799008, rs764508464, and rs754122321 that can have the opposite effect on the expression of the corresponding genes (Table 2). Using our primary keyword search, we found four articles [90–93] similar to those that were in the case of the known SNPs, where we learned about the correlations between the intensity of physiological and clinical manifestations under study [85–89] (Table 2). Finally, our secondary keyword search yielded 12 reviews [93–105], among which, the most relevant for us was the notion that *Helicobacter pylori* infection can cause not only cancer of non-reproductive organs, but can directly reduce human reproductive potential in both men and women [101].

Looking through Tables 1, 2, and Additional file 3: Table S1, one can see that a person increases his/her lifespan and reproductive potential when this person reduces the encounters with cancer risk factors.

### Known and candidate reproductivity-related SNP markers of metabolism

**Human** ***LEP*,** ***GCG*,** ***GH1*, and** ***INS*** **genes** encode hormones leptin, glucagon, somatotropin, and insulin, respectively. There are four known biomedical SNP markers: rs201381696 (obesity [54, 106]), rs183433761 (resistance to obesity during a high-fat diet [54]), rs11568827 (short stature [107]), and rs5505 (type 1 diabetes after neonatal diabetes mellitus [108]) as presented in Table 3.

Near these known SNP markers, 10 candidate SNP markers rs200487063, rs34104384, rs757035851, rs796237787, rs768454929, rs761695685, rs774326004, rs777003420, rs563207167, and rs11557611 were first predicted by our Web service [53] and, then, were characterized by our primary keyword search (Table 3). The most interesting among these predictions [109–116], in our opinion, is the candidate SNP marker rs563207167 of neonatal macrosomia whose known clinical marker is hyperinsulinemia [115], which can be caused by the minor allele of this SNP according to our calculations (Table 3).

Finally, our secondary keyword search produced 31 original articles [105, 117–146], e.g., showing that a maternal high-fat diet elevates the risk of hypertrophy in offspring via fetal hyperinsulinemia programmed epigenetically [141]. It is also relevant that bupropion used as an antidepressant against smoking in pregnancy can cause hyperinsulinemia in newborn children [142].

Human genes *NOS2*, *STAR*, *APOA1*, *CETP*, *SOD1*, *TPI1*, and *GJA5* code for inducible nitric oxide synthase 2, steroidogenic acute regulatory protein, apolipoprotein A1, cholesteryl ester transfer protein, Cu/Zn superoxide dismutase, triosephosphate isomerase, and connexin 40, respectively. Their promoters contain eight known biomedical SNP markers shown in Table 4.

Around these known biomedical SNP markers, we found six unannotated SNPs rs544850971, rs17231520, rs569033466, rs757176551, rs781835924, and rs587745372, which can alter expression levels of the human genes containing them according to *in silico* predictions of our Web service [53] (Table 4). Next, we carried out our primary keyword search where [147–165] the most interesting finding (in our opinion) is the clinical association between a SOD1 deficiency and asthenospermia [151], as one can see in Table 4. Finally, we performed our secondary keyword search, which yielded 21 literary sources [155–175]. For instance, bisphenol A pollution in men can increase the risk of congenital heart morphogenesis disorders in their offspring as Lobmo and colleagues [174] have reported.

As readers can see in Tables 3, 4, and Additional file 3: Table S1, deviations from normal metabolism in parents (e.g., starvation, stress, dietary changes, and polluted environment) can epigenetically program pathologies of the development in their offspring (e.g., [141]). Therefore, a person can increase his/her reproductive potential and lifespan by keeping one’s metabolism normal.

### Known and candidate reproductivity-related SNP markers related to blood

**Human genes** ***HBB*,** ***HBD*,** ***HBG2*,** ***ACKR1*,** ***MBL2*,** ***MMP12*, and** ***F2*** encode subunits β, δ, and γ2 (fetal) of hemoglobin as well as glycoprotein D, mannan-binding lectin, macrophage elastase, and serine protease, respectively. Table 5 shows 10 known SNP markers (rs397509430, rs33980857, rs34598529, rs33931746, rs33981098, rs34500389, and rs35518301) of both malaria resistance and thalassemia [176] as well as rs2814778 (both malaria resistance and low white-blood-cell count [177, 178]), rs72661131 (variable immunodeficiency [179], preeclampsia [180], and stroke [181]), and rs2276109 (lower risks of psoriasis [182], systemic sclerosis [183], and asthma [184]).

Using our Web service [53], we found seven candidate SNP markers rs63750953, rs281864525, rs117785782, rs34166473, rs745580140, rs562962093, and rs572527200, which can alter expression of the human genes containing them, as is the case for the above SNP markers, which can affect the human reproductive potential [185, 186] (Table 5). In addition, using our primary keyword search, we identified three more candidate SNP markers: rs567653539 (reduced risks of recurrent vulvovaginal infections [187]), rs572527200 (high risk of ovarian hyper stimulation syndrome [188]), rs564528021, and rs752364393 (high risk of pre-eclampsia [189]). Finally, we performed our secondary keyword search, which yielded 22 reviews [162, 190–210], the most important of which (in our opinion) mentions pre-eclampsia as a leading cause of maternal and fetal mortality and morbidity worldwide [162], as readers can see in Additional file 3: Table S1.

Human genes *THBD*, *PROC*, *F8*, *F3*, *F7*, *F9*, and *F11* code for thrombomodulin, and blood coagulation factors XIV, 8, 3, 7, 9, and 11, respectively (Table 6). There are three known SNP markers rs13306848 (thrombosis [211]), rs563763767 (myocardial infarction and thrombosis [212]), and F7:-33a→c (moderate bleeding [213]) located within the promoters of these genes, which are listed in Table 6.

Within 90-bp proximal regions of these promoters, we selected 30 candidate SNP markers of tumor invasion [214], thrombosis, inflammation and pregnancy loss [215–217], ovarian cancer [218], hemorrhage [219], angioneurotic edema [220], hemophilia B [221], and myocardial fibrosis [222] (Table 6). We predicted them using our Web service [53] and a primary keyword search, as described above in detail. Finally, our secondary keyword search produced 29 reviews [101, 223–250]. The most interesting among them, in our opinion, is the fact that *Homo sapiens* is the longest-lived species among great apes (*Hominidae*) in the postreproductive period. Most often, this period in the life of a human is accompanied by various types of dementia and atherosclerosis, whereas cardiomyopathy and myocardial fibrosis predominate in great apes [248].

Looking through Tables 5, 6, and Additional file 3: Table S1, readers can see that by reducing the risk of blood diseases, a person can increase his/her lifespan and reproductive potential.

### Candidate SNP markers of reproductivity-related genes

In addition, using a standard keyword search in the PubMed database, we found articles on human reproductive potential. On this basis, we selected a set of 22 human genes—*AR*, *CAT*, *CLCA4*, *CYP1B1*, *CYP17A1*, *DAZ1*, *DAZ2*, *DAZ3*, *DAZ4*, *DEFB126*, *DNMT1*, *GNRH1*, *LHCGR, MTHFR*, *NR5A1*, *PARP1*, *PYGO2*, *SRD5A2, SRY*, *TACR3*, *TET1*, and *TSSK2*—whose promoters do not contain known biomedical SNP markers. This gene set represents a wide variety of known reproductivity-related physiological markers, such as enzymes, transcription factors, hormones, and their receptors. Table 7 presents the results obtained using our Web service [53].

None of the SNPs can statistically significantly alter TBP’s affinity for the promoters of human genes *CAT*, *CLCA4*, *CYP1B1*, *DAZ1*, *DAZ2*, *DAZ3*, *DAZ4*, *DEFB126*, *GNRH1*, *LHCGR, PARP1*, *PYGO2*, *SRD5A2, SRY*, *TACR3*, *TET1*, and *TSSK2* being analyzed (data not shown). Within promoters of five remaining genes (*AR*, *MTHFR*, *DNMT1*, *CYP17A1*, and *NR5A1)*, in the same way, we found 24 candidate SNP markers (Table 7). Our primary keyword search associated them with androgenetic alopecia and androgen-induced premature senescence in adult men [251], preeclampsia [252], adverse pregnancy outcomes [253], epigenetic disorders of fetal/newborn brain development [254, 255], activation of protooncogenes in cancer [256], hyperandrogenism in polycystic ovary syndrome [257], fertility impairments [258], adrenal tumors and endometriosis [259] (Table 7).

As a cross-validation test, we unexpectedly found the ratio 5:19 of the candidate SNP markers in the reproductivity-related genes (Table 7) decreasing versus increasing TBP-promoter affinity. In contrast, the well-known whole-genome ratio 2:1 of SNPs reducing versus SNPs increasing affinity of the transcription factors for human gene promoters has been identified by two independent teams [260, 261]. According to binomial distribution, this difference between the candidate SNP markers in the reproductivity-related genes (Table 7) and all SNPs of the human genome is statistically significant (α < 0.000005). This statistical significance reflects the stronger pressure of natural selection against underexpression of the reproductivity-related genes. This unexpected finding indicates higher robustness of this specific sort of human genes on a whole-genome scale and is consistent with the commonly accepted meaning of the term “reproductive potential” as a mainstream concept in population ecology, which defines this term as a measure of evolutionary success of either human individuals [2] or populations [3]. This match between our predictions (Table 7) and one of the mainstream biomedical concepts [2, 3] support the plausibility of the candidate SNP markers predicted here.

### Verification procedures for the selected candidate SNP markers predicted here

**Fig. 2:**
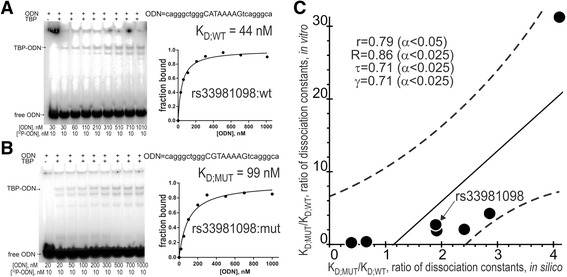
Experimental verification of the selected candidate SNP markers by an electrophoretic mobility shift assay (EMSA) *in vitro*. Legend: **a** and **b** Examples of electropherograms in the case of ancestral (panel A: norm, wild-type, wt) and minor (panel **b**: minor) alleles of the candidate SNP marker rs33981098 within the human *HBB* gene promoter and the corresponding diagrams of experimental values. **c** The significant correlations between the ratio of K_D_ values of the equilibrium dissociation constant of the TBP–ODN complex, which were either measured *in vitro* (Y-axis) or *in silico* predicted (X-axis). Solid and dashed lines or curves denote the linear regression and boundaries of its 95% confidence interval, calculated using software Statistica (Statsoft^TM^, USA). Circles denote the ancestral and minor alleles of the candidate SNP markers rs563763767, rs33981098, rs35518301, rs1143627, rs72661131, rs1800202, and rs7277748 being verified; r, R, τ, γ, and α are linear correlation, Spearman’s rank correlation, Kendall’s rank correlation, Goodman–Kruskal generalized correlation, and their significance, respectively.

Different public Web services [21–38, 53] have their advantages and disadvantages in eliminating unannotated neutral SNPs. To optimize such knowledge, a comparison between the results of these Web services and experimental data as an independent commonly accepted uniform platform seems to be a necessary step for prediction of candidate SNP markers *in silico* [15, 20, 59]. Keeping this in mind, we selected some of the 126 candidate SNP markers predicted here—rs563763767, rs33981098, rs35518301, rs1143627, rs72661131, rs1800202, and rs7277748—and measured equilibrium dissociation constant K_D_ of TBP–DNA complexes using an electrophoretic mobility shift assay (EMSA) *in vitro* (see Methods). The results are shown in Fig. 2, for example, panels A and B present electropherograms and their graphical representation in the case of ancestral and minor alleles, respectively, of the candidate SNP marker rs33981098 within the human *HBB* gene promoter. Here, readers can see that this SNP reduces the TBP–DNA affinity in half: from 44 nM in the norm (wt) to 90 nM in pathology (mut); this finding supports our prediction, namely, the twofold decrease in the estimate of TBP–DNA affinity from 5 to 9 nM (Table 5). Overall, panel C shows the coordinate plane of the predicted (axis X) and the measured (axis Y) ratio of K_D;MUT_/K_D;WT_ values of minor versus ancestral alleles of each SNP being verified. As one can see in this figure, there is a significant correlation between our predictions *in silico* and our measurements *in vitro* in four statistical tests, namely: linear correlation (r), Spearman’s rank correlation (R), Kendall’s rank correlation (τ), and Goodman–Kruskal generalized correlation (γ) test, which confirm one another’s results. Therefore, the correlations between our predictions and experimental data are robust in terms of the variation of statistical criteria that supports the candidate reproductive-potential-related SNP markers predicted here.

**Fig. 3:**
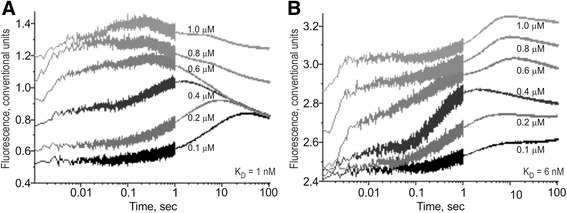
The kinetics of binding to and bending of the ODN corresponding to the selected SNP marker rs1800202. Legend: **a** The ancestral allele, ODN 5′-ctcTATATAAgtggg-3′. **b** The minor allele, ODN 5′-ctcTATAgAAgtggg-3′. ODN concentration was 0.1 μM. TBP concentration was between 0.1 and 1.0 μM as indicated near the corresponding curve of the time series. K_D_ values, **a** 1 nM and **b** 6 nM, were obtained as the output of the Dynafit software (Biokin, USA) when we used the corresponding time-series data as input for this software

Besides the conventional EMSA, we used two modern high-performance methods. Figure 3 shows the results of high-resolution spectrometry on SX.20 (Applied Photophysics, UK), where a stopped-flow fluorescence assay *in vitro* in real-time mode was applied to the selected candidate SNP marker rs1800202 (see Methods). As readers can see in Table 4, we predicted *in silico* that the K_D_ value of TBP’s binding affinity for this gene’s wild-type promoter (ancestral alleles), 1 nM, can be weakened by the minor allele of this SNP to 4 nM, in agreement with the experimental data: 1 versus 6 nM, respectively (Table 4). This is one more argument in favor of the significance of the candidate reproductive-potential-related SNP markers predicted here.

**Fig. 4:**
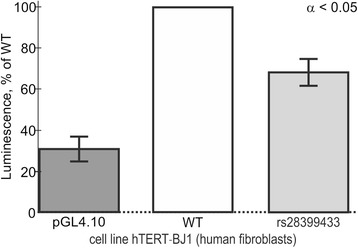
Cell culture verification of the selected candidate SNP marker rs28399433 in cell line hTERT-BJ1 (human fibroblasts) transfected with the pGL 4.10 vector carrying a reporter LUC gene. Legend: Dark gray bar, the original vector pGL 4.10 (Promega, USA) without any insertions, which served as an independent control; open bars, ancestral allele (wild type, WT); light gray bar, minor allele (rs28399433). The height of the bars and their error bars correspond to the mean estimates and boundaries of their 95% confidence intervals calculated from five independent experiments. All differences are statistically significant at the confidence level of α < 0.05

Finally, we conducted transfection of the human cell line hTERT-BJ1 (human fibroblasts) in culture, using the pGL 4.10 vector carrying a reporter LUC gene whose transcription is initiated by either ancestral or minor alleles of the selected candidate SNP marker rs28399433 of the human *CYP2A6* promoter (Table 2). The results are depicted in Fig. 4. As shown in Table 2, the low affinity of TBP for the minor allele of this SNP relative to the norm (ancestral allele) is consistent with the *ex vivo* underexpression of a reporter *LUC* gene carrying the minor allele of this SNP within the pGL 4.10 vector. This *ex vivo* observation independently confirms our prediction that this SNP can reduce the affinity of TBP for the promoter of the human *CYP2A6* gene (Table 2).

Thus, three independent experiments indicate that the candidate reproductive-potential-related SNP markers predicted here using our Web-service [53] seem to have statistically significant effects and are not neutral.

## Discussion

In this work, we limited our research to SNPs altering TBP’s affinity for human gene promoters (according to predictions made by our Web service [53]) and thereby altering the expression of these genes; this is because the TBP-binding site is the best-studied transcription-regulatory element [47]. Using our Web service [53], we analyzed over 1000 SNPs between nucleotide positions -70 and -20 upstream of more than 50 protein-coding regions documented in the Ensembl database [11] and found only 126 candidate reproductive-potential-related SNP markers (Tables 1,2, 3, 4, 5, 6 and 7). This 8-fold reduction in the number of possible SNPs can make the clinical cohort-based search for such biomedical SNP markers faster, cheaper, and more targeted, indeed.

For clinical verification of the candidate SNP markers predicted here, we heuristically set up their prioritization based on Fisher’s Z-tests as rank ρ-values from the “best” (A) to the “worst” (E) in alphabetical order (Tables 1, 2, 3, 4, 5, 6 and 7). With this in mind, our findings do not mean that all the eliminated SNPs (data not shown) cannot be considered candidate reproductive-potential-related SNP markers. This is because they may alter transcription factor-binding sites without disrupting the TBP-binding site (e.g., rs11568827, rs796237787, and rs16887226). To perform this sort of analysis for any of them, there are many public Web services [21–38] whose research capabilities may be enhanced when they are used in combination with our Web service [53].

It is also worth mentioning that 126 candidate SNP markers predicted here are whole-genome landmarks indicative of either elevated or reduced reproductive potential relative to the norm and can be expected to be present in patients as minor alleles of these SNPs [20]. For example, 10 candidate SNP markers of thrombosis (rs563763767, rs781855957, rs13306848, rs568801899, rs779755900, rs749456955, rs746842194, rs754815577, rs768753666, rs774688955) cause overproduction of coagulation inducers (Table 6). In pregnant women, Hughes syndrome provokes thrombosis with a fatal outcome, although this syndrome can be diagnosed and cured even at the earliest stages of its development [230–232] (Additional file 3: Table S1). Thus, in women carrying any of the above SNPs, preventive treatment of this syndrome [230–232] before a planned pregnancy can reduce the risk of death. Table 6 shows that seven SNPs (rs563763767, rs779755900, rs749456955, rs746842194, rs754815577, rs768753666, rs774688955) among the 10 mentioned above elevate the risk of myocardial infarction. Hence, a woman with some of these SNPs can improve her longevity by bringing her lifestyle in line with the knowledge that the risk of myocardial infarction elevates with total number of pregnancies, the age of the mother, as well as in pregnancy under the age of 20, in multiple pregnancies, in menstrual cycle irregularity, hypertension, preeclampsia, and in women smokers [233–236] (Additional file 3: Table S1).

Finally, during our keyword search in the PubMed database, we encountered a large variety of research articles, clinical cases, laboratory data, retrospective reviews, and empirical findings—on human reproductive potential in various life situations—from sociologists, geneticists, legal scholars, clinicians, bioinformaticians, pharmacists, psychologists, pedagogues, physiologists, economists, and other relevant experts such as specialists on management, insurance, environmental protection, health care, and law enforcement (Tables 1, 2, 3, 4, 5, 6 and 7, and Additional file 3: Table S1). This observation means that this vital knowledge is very much in demand for the general population, but it is too scattered for practice use. As one can see in Tables 1, 2, 3, 4, 5, 6 and 7 and Additional file 3: Table S1, 126 candidate reproductive-potential-related SNP markers predicted here may serve as valid whole-genome landmarks near which the above authors can organize their main research on how the evolutionary success of an individual [2] or a population [3] could be enhanced. Consequently, the results of these studies can be directly addressed to people who would like to change their lifestyle in view of the possible risks of diseases. This approach becomes possible within the framework of predictive-preventive personalized medicine based on the sequenced individual genomes.

## Conclusions

In keeping with Bowles’ lifespan theory [9], a large body of useful literature can be packaged into readable portions relevant to candidate reproductive-potential-related SNP markers for people who would like to reduce the risks of diseases corresponding to known alleles in own sequenced genome. After clinical validation, these candidate SNP markers may become useful for physicians (to improve treatment of patients) and for the general population (lifestyle choices improving longevity).

## Methods

### DNA sequences

We analyzed SNPs retrieved from the dbSNP database, v.147 [6] between nucleotide positions -70 and -20 upstream of the protein-coding regions documented by the Ensembl database [11] using the public Web service “UCSC Genome Browser” [12] as shown in Fig. 1a.

### Synthetic double-helical deoxyoligonucleotides (ODNs)

The ODNs identical to ancestral and minor alleles of the selected SNPs— rs563763767, rs33981098, rs35518301, rs1143627, rs72661131, rs1800202, and rs7277748—were synthesized and purified (BIOSYN, Novosibirsk, Russia).

### Preparation and purification of recombinant full-length human TBP

Recombinant human TBP (full-length native amino acid sequence) was expressed in *Escherichia coli* BL21 (DE3) cells transformed with the pAR3038-TBP plasmid (a generous gift from Prof. B. Pugh, Pennsylvania State University) as described elsewhere [262] with two modifications: the IPTG concentration was 1.0 instead of 0.1 mM, and the induction time was 3 instead of 1.5 h (for more details, see [263]).

### EMSA

The above ODNs were labeled with ^32^P on both strands by means of T4 polynucleotide kinase (SibEnzyme, Novosibirsk) with subsequent annealing by heating to 95°C (at equimolar concentrations) and slow cooling (no less than 3 h) to room temperature. Equilibrium dissociation constants (K_D_) for each TBP–ODN complex were measured using a conventional protocol [263] including titration of a fixed amount of the above-mentioned recombinant TBP, 0.3 nM, with the increasing concentrations of each ODN to reach an equilibrium, whose timing was determined independently for each ODN in advance. The binding experiments were conducted at 25°C in a buffer consisting of 20 mM HEPES-KOH pH 7.6, 5 mM MgCl_2_, 70 mM KCl, 1 mM EDTA, 100 μg/ml BSA, 0.01% of NP-40, and 5% of glycerol. The ТВР–ODN complexes were separated from the unbound ODN using an EMSA, and their abundance levels were measured. The results of these measurements were input into conventional software OriginPro 8, whose output was a K_D_ value expressed in nanomoles per liter, nM.

### Stopped-flow fluorescence measurements

The ODNs identical to both ancestral and minor alleles of the selected SNP rs1800202, (i.e., 5′-ctcTATATAAgtggg-3′ and 5′-ctcTATAgAAgtggg-3′, respectively) were labeled at their 5′-termini with fluorescent dyes TAMRA and FAM (BIOSYN, Novosibirsk, Russia). Combining a fixed concentration (0.1 μM) of ODNs with various concentrations (0.1, 0.2, 0.4, 0.6, 0.8, or 1.0 μM) of the above TBP, we analyzed six time-series of the fluorescence expressed in conventional units using high-resolution spectrometer SX.20 (Applied Photophysics, UK). The results of these measurements served as input into the Dynafit software (Biokin, USA), whose output was the above K_D_ values (for more details, see [264]).

### Cell culture, transfection, and reporter assays

Cell line hTERT-BJ1 (human fibroblasts) was cultivated in a complete medium consisting of Dulbecco’s modified Eagle’s medium/Nutrient mixture F-12 Ham, supplemented with 10% (v/v) of fetal bovine serum (Sigma), penicillin (100 U/mL), and streptomycin (100 μg/mL; BioloT). The culture was maintained at 37°C in a humidified atmosphere containing 5% of CO_2_ until the desired degree of confluence. The proximal core promoter (177 bp long) containing either the ancestral allele or minor allele of the selected candidate SNP marker rs28399433 (5′-tcaggcagTATAAAggcaaac-3′ or 5′- tcaggcagTAgAAAggcaaac-3′, respectively) was cloned into the pGL 4.10 vector (Promega, USA) and cotransfected with pRL-TK using Screen Fect A (InCella) as described elsewhere [265]. Next, the cells were cultured in 6-well plates for 24 h. Luciferase activity was determined using the Dual-Luciferase Reporter Assay Kit (Promega, USA) All the experiments were conducted five times independently at 80–85% confluence.

### DNA sequence analysis *in silico*

We analyzed DNA sequences between nucleotide positions -70 and -20 upstream of the protein-coding regions in the human genes retrieved from the human reference genome using the standard BioPerl library [266] via our Web service [53] in the case of ancestral alleles of SNPs under study, as described in Fig. 1b. In the case of minor alleles of these SNPs, we created sequences by hand using the above DNA sequences according to the description of these alleles from database dbSNP [6] as described in Fig. 1c. Next, clicking on the “Calculate” button (Fig. 1b, and c), we computed the maximal –ln(K_D_) value and its standard deviation ± ε of the affinity of TBP for the [–70; -20] region (where all the known sites are located) for both ancestral and minor alleles of the human gene promoter being analyzed. On this basis, using a package R [267], our Web service [54] made its statistical decision whether the analyzed SNP can alter the expression of the human gene under study as described in Additional file 1 [268–274]. Earlier, we tested these estimates using independent data from more than a hundred our own experiments [275–285] and the experiments of other authors (for review, see [51]). Finally, as soon as we predicted either SNP-caused significant overexpression or SNP-driven significant underexpression of the human genes being analyzed (as clinically relevant physiological markers), we conducted a manual two-step keyword search in NCBI databases [286] as described in detail in Additional file 2 [287].

## Additional files

Additional file 1: Supplementary method. A sequence-based statistical estimate of the SNP-caused alteration in the affinity of TATA box binding protein (TBP) for the human gene promoter containing this SNP within its region [-70; -20]. (PDF 220 kb)

Additional file 2: Supplementary method. Keyword search in the PubMed database. (PDF 221 kb)

Additional file 3: Table S1. Clinically known dependences between reproductive potential and hereditary diseases whose SNP markers were predicted in this work. (PDF 198 kb)

## Abbreviations

*ACKR1*: atypical chemokine receptor 1
*APOA1*: apolipoprotein A1
*AR*: androgen receptor
*CAT*: catalase
*CETP*: cholesteryl ester transfer protein
*CLCA4*: chloride channel accessory 4
*CYP17A1*: cytochrome p450 family 17 subfamily A member 1
*CYP1B1*: cytochrome P450 family 1 subfamily B member 1
*CYP2A6*: cytochrome P450 family 2 subfamily A member 6
*CYP2B6*: cytochrome P450 family 2 subfamily B Member 6
*DAZ1 (2, 3, 4)*: deleted in azoospermia 1 (2, 3, 4, respectively)
*DEFB126*: defensin β 126
*DHFR*: dihydrofolate reductase
*DNMT1*: DNA methyltransferase 1
EMSA: electrophoretic mobility shift assay
*ESR2*: estrogen receptor 2
*F2 (3, 7, 8, 9, 11)*: coagulation factor II (III, VII, VIII, IX, XI, respectively)
*GCG*: glucagon
*GH1*: growth hormone 1
*GJA5*: gap junction protein α5
*GNRH1*: gonadotropin releasing hormone 1
*GSTM3*: glutathione S-transferase μ3
*HBB*: hemoglobin subunit β
*HBD*: hemoglobin subunit δ
*HBG2*: hemoglobin subunit γ2
*HSD17B1*: hydroxysteroid 17-β dehydrogenase 1
*IL1B*: interleukin 1 β
*INS*: insulin
K_d_: equilibrium dissociation constant
*LEP*: leptin
*LHCGR*: luteinizing hormone (choriogonadotropin receptor)
Ln: natural logarithm
*MBL2*: mannose binding lectin 2
*MMP12*: matrix metallopeptidase 12
*MTHFR*: methylenetetrahydrofolate reductase
Mut: minor allele of SNPs. Genes
*NOS2*: nitric oxide synthase 2
*NR5A1*: nuclear receptor subfamily 5 group A member 1
*PARP1*: poly(ADP-ribose) polymerase 1
*PGR*: progesterone receptor
*PROC*: protein C (inactivator of coagulation factors Va and VIIIa)
*PYGO2*: pygopus family PHD finger 2
SNP: single nucleotide polymorphism
*SOD1*: superoxide dismutase 1
*SRD5A2*: steroid 5 α-reductase 2
*SRY*: sex determining region Y
*STAR*: steroidogenic acute regulatory protein
*TACR3*: tachykinin receptor 3
TBP: TATA-binding protein
*TET1*: Tet methylcytosine dioxygenase 1
TF: transcription factor
*THBD*: thrombomodulin
*TPI1*: triosephosphate isomerase 1
TSS: transcription start site
*TSSK2*: testis specific serine kinase 2
WT: wild type (norm)
