# Supplementary material for: Candidate SNP markers of reproductive potential are predicted by a significant change in the affinity of TATA-binding protein for human gene promoters
Source: BMC Genomics. 2018 Feb 9;19(Suppl 3):0. doi: 10.1186/s12864-018-4478-3 (PMC5836831; doi:10.1186/s12864-018-4478-3)
Supplement: Supplementary file 1 — Supplementary method. A sequence-based statistical estimate of the SNP-caused alteration in the affinity of TATA box binding protein (TBP) for the human gene promoter containing this SNP within its region [-70; -20]. (PDF 220 kb) [file 12864_2018_4478_MOESM1_ESM.pdf]

Additional file 1: Supplementary Method.

A sequence-based statistical estimate of the SNP-caused alteration in the affinity of TATA-box binding protein (TBP) for the human gene promoter containing this SNP within its region [–70; –20]

The initializing data are the DNA sequence $\{s_{-90}\ldots s_i\ldots s_{-1}\}$ of 90 bp in length that is located upstream of the transcription start site (TSS) of a given gene in humans and a given single nucleotide polymorphism (SNP) of this DNA sequence whose ancestral (wt) and minor (mut) alleles are compared with each other (where s_0 = TSS).

First, we calculate the $-\ln(K_D)$ value of the TBP–promoter affinity for the ancestral allele of this sequence:

$$\begin{cases} -\boldsymbol{ln}(\boldsymbol{K_D}) = \mathbf{10.9} - \mathbf{0.2}\{\boldsymbol{ln}(\boldsymbol{K_{D;SLIDE}}) + \boldsymbol{ln}(\boldsymbol{K_{D;STOP}}) + \boldsymbol{ln}(\boldsymbol{K_{D;BEND}})\}; & (1) \\ -\boldsymbol{ln}(\boldsymbol{K_{D;SLIDE}}) = \underset{\substack{15-bp\ window \\ (+),(-)\ DNA\ chains}}{\mathbf{MEAN}} \left\{\mathbf{0.8}[\boldsymbol{TA}]_{\boldsymbol{3'HALF}} - \mathbf{3.4}\underset{CENTER}{\mathbf{MEAN}}(\textbf{minor groove width}) - \mathbf{35.1}\right\}; & (2) \\ -\boldsymbol{ln}(\boldsymbol{K_{D;STOP}}) = \underset{\substack{i\in[-70;\,-20] \\ (+),(-)\ DNA\ chains}}{\mathbf{MAX}} \left\{\sum_{j=-1}^{13} \boldsymbol{w_{j;s_{i+j}}}\right\}; & (3) \\ -\boldsymbol{ln}(\boldsymbol{K_{D;BEND}}) = \underset{\substack{TATA-box\ (Eq.(3)) \\ (+),(-)\ DNA\ chains}}{\mathbf{MEAN}} \{\mathbf{0.9}[\boldsymbol{WR} \in \{\boldsymbol{TA,AA,TG,AG}\}]_{\boldsymbol{3'HALF}} + \mathbf{2.5}[\boldsymbol{TV} \in \{\boldsymbol{TA,TG,TC}\}]_{\boldsymbol{CENTER}} + \mathbf{14.4}\}. & (4) \end{cases}$$

Here, K_D is the equilibrium dissociation constant of a given TBP–TATA complex, expressed in moles per liter (M). Eq. (1) is the linear approximation of three steps of TBP's binding to the [–70; –20] promoter region upstream of the TSS being studied. These steps are (i) TBP slides along DNA, (ii) TBP stops at a potential TATA box met, and the DNA helix bends to the 90° angle in order to stabilize the complex between TBP and this TATA box [279]. 10.9 (ln units) is nonspecific TBP–DNA affinity (10^{-5} M [280]). 0.2 is the stoichiometric coefficient [279]. $K_{D;SLIDE}$ is our heuristic estimate of an impact of TBP's sliding along DNA on the above K_D value using Eq. (2) [281]. $[TA]_{3'HALF}$ is the frequency of dinucleotide TA within the 3′ half of the promoter under study [281]. MGW_{CENTER} is the arithmetical mean width of the minor groove of the DNA helix [282]; 0.8, –3.4, and –35.1 are linear regression coefficients taken from our original experimental data [281]. K_{STOP} is our heuristic estimate of the impact of TBP's stoppage at a potential TATA-box recognized using the commonly accepted criterion based on the maximal score value of Bucher's position-weighted matrix [283]. K_{BEND} is our heuristic estimate of the impact of the DNA helix bend [284] according to our original experimental data [285]. 0.9, 2.5, and 14.4 are linear regression coefficients.

Next, we estimate the standard error $\pm\varepsilon$ of the above $-\ln(K_D)$ value in the standard manner according to all the possible alterations of this $-\ln(K_D)$ value corresponding to single nucleotide substitutions within the 26-bp DNA window around the above TATA-box matching Bucher's criterion [283], Eq. (3) as shown above.

Then, in the same way, we calculate the same $-\ln(K_D) \pm \varepsilon$ values for the minor allele of the SNP being studied.

Thus, two pairs of values "$-\ln(K_D^{(wt)}) \pm \varepsilon_{(wt)}$" and "$-\ln(K_D^{(mut)}) \pm \varepsilon_{(mut)}$" corresponding to the above-mentioned ancestral and minor alleles are obtained, and we calculate Fisher's Z-score, which characterizes a difference between them, as

$$\boldsymbol{Z} = \frac{\left|\boldsymbol{ln}\left(\boldsymbol{K_D^{(wt)}}/\boldsymbol{K_D^{(mut)}}\right)\right|}{\sqrt{\boldsymbol{\epsilon^2_{(wt)}}+\boldsymbol{\epsilon^2_{(mut)}}}}. \qquad (5)$$

Thus, we convert this Z-score value into the p value of the probability rate of acceptance of the hypothesis "H_0: $-\ln(K_D^{(wt)}) \neq -\ln(K_D^{(mut)})$" using statistical package R [278] (where $\alpha = 1 - p$ is the statistical significance).

Finally, we made the final decision at the proper statistically significant level $\alpha < 0.05$ (i.e., at $p > 0.95$), as follows:

IF {**INEQUALITY** "$-\ln(K_D^{(mut)}) > -\ln(K_D^{(wt)})$" is statistically significant},

THEN {**DECISION** is "the minor allele of a given gene is overexpressed relative to norm (ancestral allele)"};

ELSE [**IF** {**INEQUALITY** "$-\ln(K_D^{(mut)}) < -\ln(K_D^{(wt)})$" is statistically significant},

THEN {**DECISION** is "the minor allele of a given gene is underexpressed relative to norm (ancestral allele)"}]

OTHERWISE {**DECISION** is "alteration of the expression of this gene is insignificant"}.

This DECISION is the sequence-based statistical estimate of the SNP-caused alteration in the affinity of TBP for the human gene promoter containing this SNP in the [–70; –20] region of this promoter. This is the public Web service SNP_TATA_Comparator [53], "http://beehive.bionet.nsc.ru/cgi-bin/mgs/tatascan/start.pl".
