# Supplementary material for: Candidate SNP markers of reproductive potential are predicted by a significant change in the affinity of TATA-binding protein for human gene promoters
Source: BMC Genomics. 2018 Feb 9;19(Suppl 3):0. doi: 10.1186/s12864-018-4478-3 (PMC5836831; doi:10.1186/s12864-018-4478-3)
Supplement: Supplementary file 2 — Supplementary method. Keyword search in the PubMed database. (PDF 221 kb) [file 12864_2018_4478_MOESM2_ESM.pdf]

Additional file 2: Supplementary Method.

Keyword search in the PubMed database

We conducted a manual two-step keyword search in NCBI databases [298] as described in detail elsewhere [299] and as depicted schematically in Figure S1 as soon as we predicted either SNP-caused significant overexpression or SNP-caused significant underexpression of the human genes being analyzed (as clinically relevant physiological markers).

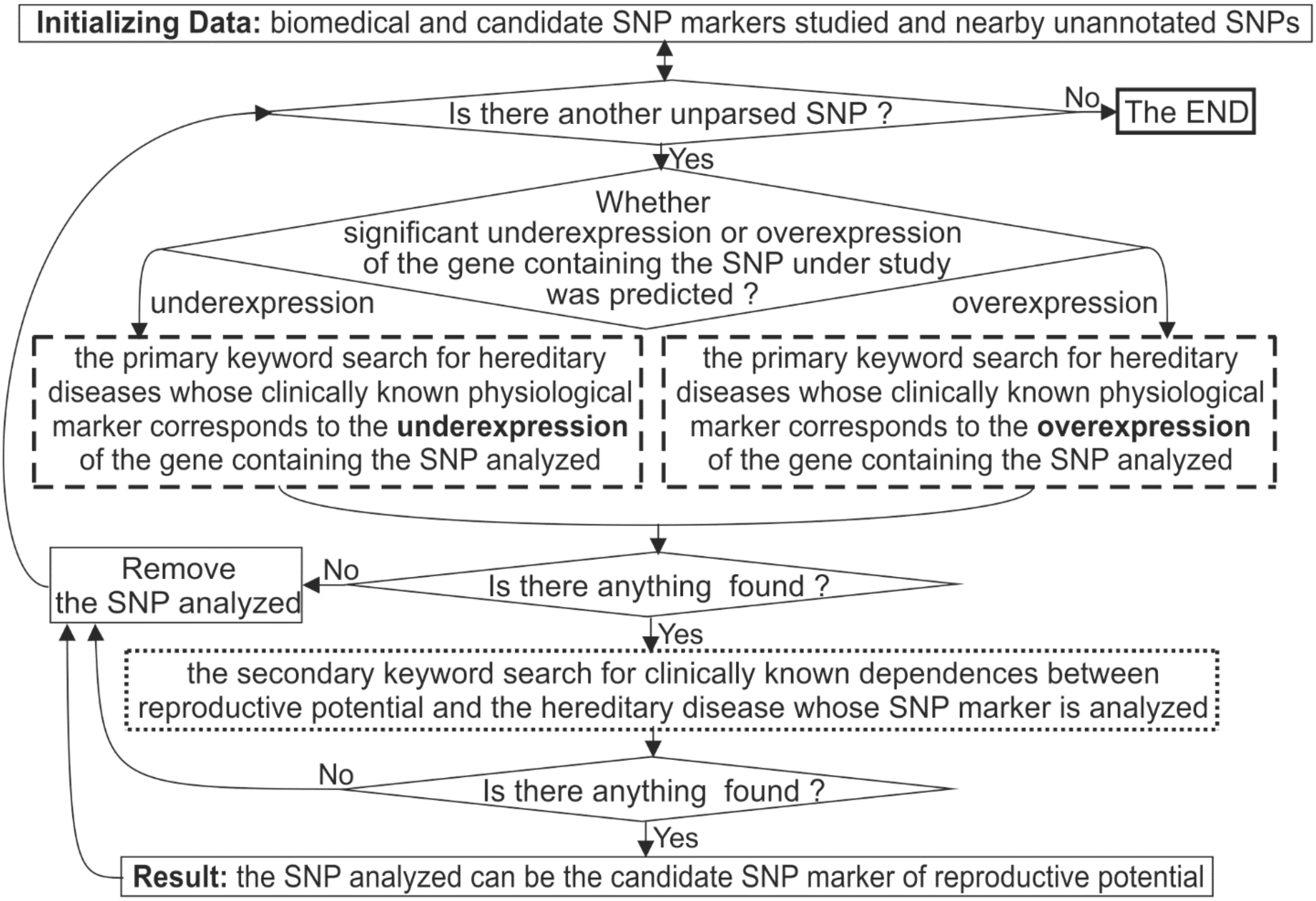

Figure S1. A flow chart of the keyword search for reproductive potential whose physiological markers correspond to an alteration in expression of the human gene under study containing the candidate SNP marker of interest.

Legend. Two dashed-line boxes depict the primary keyword search for hereditary diseases whose clinically known physiological marker corresponds to either under- or overexpression of the human gene with the minor allele of the SNP being analyzed relative to the norm (ancestral alleles). The dotted-line box depicts the secondary keyword search for clinically known dependences between reproductive potential and the disease found whose SNP marker is being analyzed.

In this figure, two dashed-line boxes depict the primary keyword search for diseases whose symptoms affect reproductive potential and whose known physiological markers correspond to the gene expression alteration caused by the SNP being considered.

In addition, in Fig. S1, the dotted-line box depicts the secondary keyword search for clinically known dependences between reproductive potential and the hereditary disease found above whose SNP marker is being analyzed.

The results obtained using our primary and secondary keyword searches and our heuristic interpretations of these data are in the second rightmost column of Tables 1–7 (Results and Discussions) and S1 (Additional file 3) where they are *italicized* and preceded by the label "*(hypothetically)*." In addition, all the literary sources found are cited in the rightmost columns of Tables 1–7 and S1 where they are also *italicized* and preceded by another label "*[this work]*."
