# Supplementary material for: Candidate SNP markers of reproductive potential are predicted by a significant change in the affinity of TATA-binding protein for human gene promoters
Source: BMC Genomics. 2018 Feb 9;19(Suppl 3):0. doi: 10.1186/s12864-018-4478-3 (PMC5836831; doi:10.1186/s12864-018-4478-3)
Supplement: Supplementary file 3 — Table S1. Clinically known dependences between reproductive potential and hereditary diseases whose SNP markers were predicted in this work. (PDF 198 kb) [file 12864_2018_4478_MOESM3_ESM.pdf]

Additional file 3: Supplementary Table S1**.**

Table S1: Clinically known dependences between reproductive potential and hereditary diseases whose SNP markers were predicted in this work.

Candidate SNP-marker, dbSNP ID [6]	Hereditary diseases, drugs	*Clinically known dependences between reproductive potential and a hereditary disease whose SNP is being analyzed*	*[this work], [Ref]*
rs35036378, rs766797386	ESR2-deficient pT1 tumor	*Cadmium (Cd) as a carcinogen elevates risks of both a benign tumor and malignant transformation*	*[62]*
	tamoxifen	*women undergoing tamoxifen treatment should not breastfeed*	*[63]*
rs201739205, rs201739205, rs748743528, rs755636251	breast cancer	*there is a nontrivial balance between reproductive ability and risk of cancer of reproductive organs; the only known so far SNP marker rs605059 within the protein-coding region of the human HSD17B1 gene increases both lifespan and number of children in women*	*[70, 71]*
rs10895068	endometrial cancer	*early childbearing can best realize reproductive potential because early endometrial cancer treatment in a young woman offers less than a 60% chance of keeping fertility*	*[72]*
rs1332018, rs200209906, rs750789679, rs748231432, rs763859166	brain tumor	*when any cancer metastasizes to the brain, breast cancer does it more;<br>Cowden's syndrome reliably often causes both brain and breast cancers in women.*	*[73, 74]*
	Alzheimer's disease	*one of current theories is that aging is a stepwise reduction in reproductive potential of individuals where one of these steps is under the control of the luteinizing hormone, whose suppression by smoking can reduce risk of Alzheimer's disease*	*[9]*
	renal cancer	*estrogen treatment elevates risk of renal cancer; one or two children born by a woman reduce risk of renal cancer in this woman relative to the cases of three or more and lack of children, as well as the risk of renal cancer decreases with an increase in lifetime duration of ovulation; when cancer is the 2nd leading cause of death in women of childbearing age, the renal cancer seems to be the rarest tumor they have.*	*[75, 76]*
rs28399433, rs761592914	smoking (and lung tumors)	*smoking and secondary smoke increase mother's bodyweight and risk of preterm delivery in underage mothers, as well as they decrease both baby's bodyweight and height*	*[94]*
rs34223104, rs563558831	bioactivation of antitumor pro-drug cyclophosphamide	*cyclophosphamide has a high gonadal toxicity, up to a high risk of premature gonadal failure (as a complication of either antitumor and antiautoimmune treatments based of a cyclophosphamide use, especially in the case of female patients) that can be reduced due to an adjuvant hormonal therapy based on the estrogen-containing oral contraceptives for females and testosterone for males.*	*[95, 96]*
rs10168, rs750793297, rs766799008, rs764508464, rs754122321	leukemia	*for antileukemia treatment now, the only conventional way of fertility preservation is cryopreservation of sperm, oocytes, and embryos together with gonadal shielding and transposition of ovaries during irradiation.*	*[97]*
	methotrexate	*methotrexate-based therapy for ectopic pregnancies, metastatic choriocarcinoma, and gestational trophoblastic disease;<br>men should avoid methotrexate for three months before planned conception*	*[98, 99]*
rs1143627, rs549858786	non–small cell lung cancer	*lung cancer risk decreases with the estrogen plus progestin use for at least 5 years as well as with increasing age at menopause and, in the only case of non–small cell lung cancer, together with age 20–29 years at first live birth, but there is a trend toward increased risk of lung cancer with the increasing number of live births.*	*[100]*
	liver cancer	*late age at menarche and even use of menopausal hormonal therapy reduce risk of primary liver cancer; oophorectomy increases risk of primary liver cancer*	*{101]*
	gastric cancer and ulcer, chronic gastritis	*Helicobacter pylori infection reduces human reproductive potential in both men and women*	*[102]*
	Graves' disease	*Graves' disease as maternal thyroid dysfunction may cause both pregnancy complications and developmental defects in either fetus or child.*	*[103]*
	recurrent major depression	*high doses of exogenous oral estrogen just after birth reduce from 60% to 9% risks of both puerperal acute affective psychosis and early-onset recurrent major depression in mentally healthy women*	*[104]*
rs200487063	obesity	*female obesity is associated with greater risk of higher rates of infertility, subfertility, early pregnancy loss, fetal deaths and stillbirths, congenital anomalies, and pregnancy complications, including prematurity and pre-eclampsia; obesity elevates both serum and seminal levels of both insulin and leptin, as well as both sperm mitochondrial membrane potential and DNA fragmentation; seminal insulin and leptin that reduces sperm vitality and reproductive potential in men without visible changes in sperm motility and morphology.*	*[105, 106]*

Table S1: continued

Candidate SNP-marker, dbSNP ID [6]	Hereditary diseases, drugs	*Clinically known dependences between reproductive potential and a hereditary disease whose SNP is being analyzed*	*[this work], [Ref]*
rs200487063	hypo-leptinemia	*reduced fertility in men, lactation failure, for the treatment of which leptin replacement therapy is used; delayed menarche because of up to 16 kg threshold of fat needs to be accumulated during childhood until puberty in the norm, whose indicator is adipocyte lectin, to have enough energy for each pregnancy and lactation pair; higher risk of infertility because of anorexia nervosa*	*[118, 121]*
rs34104384, rs201381696	hyper-leptinemia	*advanced puberty, ovarian disorders, embryogenesis disorders, reduced fetal viability, decreased testosterone level; accelerated menarche; maternal high-fat diet during pregnancy and lactation elevates risks of higher birth weight, fat accumulation and hyperleptinemia in new birth; maternal undernutrition and starvation during pregnancy elevates risks of low birth weight as well as obesity, hyperleptinemia, and premature senescence in adult offspring; hyperleptinemia is a biomarker of childhood neurodevelopmental disorders including autism; hyperleptinemia is a biomarker of polycystic ovary syndrome; hyperleptinemia elevates risks of pre-eclampsia in hypertensive pregnancy*	*[118, 122-126]*
	hypertension in obesity	*prepregnancy obesity, excessive gestational weight gain, and increased intake of energy elevates risk of gestational hypertension's causing a significant part of maternal, fetal, and neonatal mortality and morbidity in the world; polycystic ovary syndrome elevates risks of diabetes, obesity, and hypertension; maternal prepregnancy obesity and excessive gestational weight gain can promote fetal hyperlectinemia during a critical window of hypothalamic development that may directly lead to early-adulthood hypertension in offspring; environmental risk factors of adult hypertension initiated in fetal life are calorie excess (obesity and alcohol intake), high salt intake, low potassium intake, physical inactivity, and psychosocial stress.*	*[127-130]*
rs183433761, rs757035851	hypo-glucogonemia	*reduced pancreatic glucagon in infants of diabetic mothers elevates risk of hypoglycemia*	*[131]*
rs11568827, rs796237787, rs768454929, rs761695685	short stature	*in pediatric patients with idiopathic short stature and those born small for gestational age, as well as in the case of GH1-deficiency, the treatment based on recombinant somatotropin allows to increase adult height.*	*[132]*
	GH1-deficiency	*GH1 deficiency can modify folliculogenesis, ovarian maturation, ovulation, pregnancy, sexual maturation, the menstrual cycle, and the reproductive ability in women, as well spermatogenesis and the Leydig cell function in men; high risks of hyperthermia during physical activity in a hot environment.*	*[133-135]*
rs774326004, rs777003420	acromegaly	*gonadal dysfunction is very common in premenopausal women with acromegaly.*	*[136]*
rs11557611	hypo-insulinemia	*reproductive behavior deficiency in diabetic men is caused by hypoinsulinemia via hyperglycemia rather than by hyperglycemia directly; prenatal hypoinsulinemia can result in slower behavioral development of the newborns when weight first grows slower, elementary behavioral acts next appear later, and, finally, complex behavioral patterns are formed later; hypoinsulinemia can cause asymmetric intrauterine growth retardation; hypoinsulinemia is a biomarker of the human status during lactating along with prolonged hyperglycemic stimulation; glucose as an energy source against starvation in pregnancy can only complicate both mother and fetus states because hypoinsulinemia is a biomarker of this fasting*	*[137-141]*
rs5505, rs563207167	hyper-insulinemia	*hyperinsulinemia is very frequent in pregnancy; obesity reduces reproductive potential in men via hyperinsulinemia and hyperleptinemia; maternal high-fat diet causes fetal hyperinsulinemia, which in turn causes cardiac hypertrophy in offspring; bupropion as an antidepressant against smoking during pregnancy can cause severe hyperinsulinemia in newborns; hyperinsulinemia as a biomarker of familial predisposition to polycystic ovary syndrome*	*[106, 140, 142-144]*
NOS2: -51t→c [148]	gestational diabetes mellitus<br>epilepsy	*gestational diabetes mellitus is the statistically significantly frequent complication of pregnancy in women with epilepsy after cesarean deliveries and congenital malformations; preparing for pregnancy and its planning together with an epileptologist allows to control seizures using minimal doses of antiepileptic drugs and minimizes risks of complications of both pregnancy and delivery.*	*[145-147]*
rs16887226	hypertensive diabetes	*the higher rate of induction of labor differs only between hypertensive and nonhypertensive diabetic pregnancies*	*[167]*
APOA1: –35a→c [152]	fatty liver	*nonalcoholic fatty liver as the common liver disease in the world elevates risk of adverse pregnancy outcomes regardless body–mass index and diabetes in women prior to giving birth; acute fatty liver of pregnancy is a fatal complication with jaundice, nausea, vomiting, malaise, ascites, renal failure, hepatic encephalopathy, hypersensitivity to infection, and postpartum hemorrhage so that either early termination of pregnancy or cesarean delivery are used to save women.*	*[168, 169]*
	polycystic ovary syndrome	*low percentage of natural killer cells in the secretory endometrium is indicative of infertile women with polycystic ovary syndrome; in obese infertile women with polycystic ovary syndrome, lifestyle modifications without rapid weight loss lead to a reduction of central fat and to improved insulin sensitivity that restores their ovulation and reproductive ability.*	*[170, 171]*

Table S1: continued

Candidate SNP-marker, dbSNP ID [6]	diseases, drugs	*Clinically known dependences between reproductive potential and a hereditary disease whose SNP is being analyzed*	*[this work], [Ref]*
CETP: DEL-51(18bp) [153], rs17231520, rs569033466, rs757176551	atherosclerosis	*shorter lactation duration is associated with atherosclerosis whereas lactation may have long-term benefits that lower cardiovascular disease risk in women; the higher the total number of children, the higher is the risk of carotid atherosclerosis in both younger and older women, but not among men; pre-eclampsia as a leading cause of maternal and fetal mortality and morbidity worldwide, e.g., atherosclerosis in the elderly because of oxidative stress and endothelial dysfunction in their pathophysiology as well as their common risk factors; the prodromal stage of atherosclerotic plaques is already present during human fetal development, and the plaques grow depending first on maternal hypercholesterolemia and, later, on infections throughout life.*	*[172-175]*
rs7277748	amyotrophic lateral sclerosis	*amyotrophic lateral sclerosis is a rare complication of pregnancy, whose postpartum treatment is based on bilateral autologous stem cell transplant into the frontal motor cortices*	*[176]*
	progesterone deficiency	*lower chance for normal embryo implantation and growth;<br>higher risk of recurrent miscarriage*	*[177, 178]*
	asthenospermia	*asthenospermia is a critical factor in male fertility whereas medium enriched with H_2 molecules increases the sperm mobility, selectively reduces the cytotoxic reactive oxygen species in them and improves the permeability of their mitochondrial membranes for ATP*	*[179]*
rs1800202, rs781835924	neuromuscular diseases	*neuromuscular diseases can cause complications for pregnancy development as well as for both fetus development and delivery, and these problems should be taken into account both during planning and monitoring of pregnancy and when choosing either vaginal or Cesarean delivery*	*[180]*
	hemolytic anemia	*microangiopathic hemolytic anemia is 1 of 3 obligatory symptoms of HELLP syndrome as a dangerous complication of pregnancy after 35 weeks, which can lead to hemorrhages, disseminated intravascular coagulation and eclampsia*	*[181]*
	arrhythmia	*maternal arrhythmias can complicate pregnancy when their correction by medication (especially in the first trimester) and pacemaker (due to the effects on the fetus) are limited so that smoking, caffeine and alcohol as arrhythmia provocateurs should be limited for mothers, as well as cocaine should be limited for mothers to reduce the risk of fetal arrhythmias.*	*[182, 183]*
rs10465885, rs35594137, rs587745372	cardiovascular events	*hypertensive pregnancy elevates risks of future hypertension and cardiovascular disease in both mothers and their newborn children*	*[184]*
	heart morphogenesis disorders	*small ratio of the head circumference to the body weight in the newborns elevates risk of some congenital heart morphogenesis disorders; bisphenol A pollution in men increases the risk of congenital heart morphogenesis disorders in their offspring; in congenital heart morphogenesis disorders, improvements in in-utero hemodynamics and cerebral oxygen delivery can enhance fetal brain development.*	*[185-187]*
rs397509430, rs33980857, rs34598529, rs33931746, rs33981098, rs34500389, rs35518301, rs63750953, rs281864525, rs117785782, rs34166473, rs745580140	thalassemia	*later menarche in girls with thalassemia than without it; amenorrhea in thalassemia is more frequent than the norm as well as higher risk of hypogonadotrophic hypogonadism, which is correctable by hormone replacement therapy when there are no contraindications for it..*	*[202]*
	malaria resistance	*during pregnancy and postpartum, malaria significantly correlates with high morbidity and mortality for both the mother and the fetus.*	*[203]*
rs2814778	low white-blood-cell count	*low white blood cell count is a marker of the high risk of preterm delivery;<br>maternal low white blood cell count is associated with both poor early weight gain in the baby and later retinopathy of prematurity.*	*[204, 205]*
rs564528021, rs752364393	pre-eclampsia	*pre-eclampsia is a leading cause of maternal and fetal mortality and morbidity worldwide; pre-eclampsia is associated with adverse neonatal outcomes; dietary and lifestyle changes have a potential to reduce the risk of pre-eclampsia; selenium (Se)-rich diet reduces the pre-eclampsia risk in women with low Se status; higher allostatic load caused by either repeated or chronic stress in early pregnancy is associated with increased risk of pre-eclampsia; low maternal socioeconomic status is a strong risk factor of pre-eclampsia; pre-eclampsia is associated with stage B heart failure, hypertension and higher cardiovascular risk within few years postpartum; paradoxically, smoking during pregnancy is associated with a reduced risk of any type of pre-eclampsia; obesity in prepregnant women reduces risk of pre-eclampsia in these women during pregnancy; physical activity at work increases the risk of pre-eclampsia.*	*[174, 206-214]*
rs72661131, rs562962093, rs567653539	variable immune-deficiency	*low fertility relative to the norm, in part because of the insufficient consultation regarding the possibility of pregnancy without incident and with live birth due to the newest IgG replacement therapy*	*[215, 216]*
	stroke	*pregnancy-related ischemic stroke significantly increases both maternal and fetal morbidity and mortality; pregnancy-caused hypertension elevates risk of stroke in the year following delivery; as secondary stroke prophylaxis after primary one, clopidogrel treatment before conception and up to a week before the induction of labor, as well as 12 hours after birth, prevents complications in both mother and newborn; perinatal hypoxia as one of the causes of neonatal arterial ischemic stroke; maternal hypertension and intrapartum fever appear to be risk factors of ischemic stroke in infants; pregnancy elevates risk of both ischemic stroke and intracerebral hemorrhage in young women.*	*[217-222]*

Table S1: continued

Candidate SNP-marker, dbSNP ID [6]	Hereditary diseases, drugs	*Clinically known dependences between reproductive potential and a hereditary disease whose SNP is being analyzed*	*[this work], [Ref]*
rs528817178, rs539608065, rs539731824, rs756414294, rs777687270, rs746382956	tumor cell invasion	*reproductive cancers are a major cause of cancer death in women worldwide; postpartum involution of breasts increases the lymphatic-vessel density and prolymphangiogenic activity of normal tissue relative to the same of nulliparous women, and this condition can promote the peripheral lymphatic system development, tumor growth, and tumor cell invasion up to the breast cancer onset and metastasis as a postpartum complication; early pregnancy reduces the risk of breast cancer owing to age-related changes in collagen 1 structure from high-density nonfibrillar antitumor packaging to low-density linear fibrils fitting the tumor cell invasion*	*[235-237]*
rs542626506, rs61731661	inflammation	*S. marcescens as intraperitoneal infection reduces reproductive potential in men; CagA+ strains of H. pylori reduce reproductive potential in men; male accessory gland inflammation reduces reproductive potential up to secretory dysfunction of these glands; salpingitis caused by either sexually transmitted pathogens or by abortion vis-a-vis unwanted pregnancy elevates risk of infertility up to 25% in women of 20–25 years old*	*[102, 238 - 241]*
rs781855957<br>rs13306848, rs568801899	thrombosis	*Hughes syndrome-associated thrombosis elevates risk of death during pregnancy, which is an early detectable syndrome easily preventable by medication*	*[242 - 244]*
rs563763767, rs779755900, rs749456955, rs746842194, rs754815577,	myocardial infarction	*after myocardial infarction, any woman needs medical help to restore her sexual health; myocardial infarction as life-threatening complication of pregnancy, whose risk increases with maternal age, smoking, hypertension, twin pregnancy, pre-eclampsia as well as with a lifelong irregular menstrual cycle pattern, total number of livebirths, miscarriages, or induced abortions, first pregnancy or childbirth occurred before age 20 y.o., a younger age of first childbirth in women younger than 55 y.o.*	*[245 - 248]*
rs768753666, rs774688955	ovarian cancer	*Fertility-sparing surgical treatment of ovarian cancer can keep the ability and reduces reproductive potential;*	*[249]*
F7: -33a→c [225]<br>rs749691733, rs367732974, rs549591993, rs777947114, rs770113559, rs754814507<br>rs754739433, rs780731761, rs747652067	moderate bleeding (spontaneous hemorrhage)	*intermenstrual bleeding decreases the odds of conceiving in that cycle without a negative impact on a woman's reproductive potential; bilateral hypogastric artery ligation for control of pelvic uncontrollable hemorrhage allows to keep reproductive potential even in cases such as cervical pregnancy, placenta previa, placental abruption, uterine atony, and uterine rupture; uterine compression sutures against severe postpartum hemorrhage allow to keep a woman's fertility, including uncomplicated future pregnancies that occur within a range of 1–3 years, though long-term follow-up is recommended according to potential risks of ischemic necrosis; pubertal development in girls of the current generation is much earlier, longer and with wide irregularity of menstrual cycles, the absence of adolescent bleeding at the age of 14 and its frequency out of the range of 20–45 days still indicate higher risk of reproductive failure in adulthood; both oral contraceptives and hormone replacement therapy significantly alter menstrual bleeding patterns up to amenorrhea as reproductive status.*	*[250 - 254]*
rs374761594, rs759231858, rs752308147	angioneurotic edema	*since angioneurotic edema (especially laryngeal edema) can cause death of anesthesia during caesarean and normal labor, pregnancy needs preliminary preparation at several days before labor.*	*[255-257]*
rs750827465	myocardial fibrosis	*prenatal nicotine exposure can elevate risk of myocardial fibrosis in adult male offspring; maternal inflammation (as prenatal exposure to lipopolysaccharide) can elevate risk of myocardial fibrosis in old age of offspring; humans are more likely to live long after reproductive state with dementia and coronary atherosclerosis as causes of death in the elderly, whereas cardiomyopathy and myocardial fibrosis predominate in apes.*	*[258-260]*
rs371045754	hemophilia B	*caesarean delivery is best for a hemophilia carrier expecting an affected infant; hemophilia and related factors in the family have a significant influence on women's reproductive choices.*	*[261, 262]*
